# Loneliness and social isolation is associated with sleep problems among older community dwelling women and men with complex needs

**DOI:** 10.1038/s41598-021-83778-w

**Published:** 2021-03-01

**Authors:** Laurie McLay, Hamish A. Jamieson, Karyn G. France, Philip J. Schluter

**Affiliations:** 1 School of Health Sciences, University of Canterbury – Te Whare Wānanga o Waitaha, Private Bag 4800, Christchurch, 8140 New Zealand; 2 Department of Medicine, University of Otago, Christchurch, New Zealand; 3 Canterbury District Health Board, Older Person’s Health Christchurch, Christchurch, New Zealand; 4 School of Clinical Medicine, Primary Care Clinical Unit, The University of Queensland, Brisbane, Australia

**Keywords:** Risk factors, Signs and symptoms

## Abstract

Sleep problems, loneliness and social isolation often increase with age, significantly impacting older adults’ health and wellbeing. Yet general population health empirical evidence is surprisingly scant. Using the largest national database to date, cross-sectional and longitudinal analyses was undertaken on 140,423 assessments from 95,045 (women: 61.0%) community living older adults aged ≥ 65 years having standardised home care assessments between 1 July 2012 and 31 May 2018 to establish the prevalence and relationships between insufficient sleep, excessive sleep, loneliness and social isolation. At first assessment, insufficient sleep (women: 12.4%, men: 12.7%) was more commonly reported than excessive sleep (women: 4.7%, men: 7.6%). Overall, 23.6% of women and 18.9% of men reported feeling lonely, while 53.8% women and 33.8% men were living alone. In adjusted longitudinal analyses, those who were lonely and socially isolated were more likely to experience insufficient sleep. Respondents with excessive sleep were more likely to live with others. Both loneliness and social isolation contributed to insufficient sleep, synergistically. Loneliness, social isolation and health-concerns may affect the restorative properties of sleep over and above the effects of ageing. Further research is warranted.

## Introduction

Sleep is a fundamental biological process, essential in determining human health and performance. Yet, between 10–30% of people in developed nations now experience chronic insomnia due to factors including electric lighting, shift work, increasing travel, and our digital revolution; in direct contrast to the 1.5–2.5% reported in traditional living indigenous groups within Africia^1^. Suboptimal sleep has been associated with increased mortality and morbidity as well as a number of comorbid physical and mental health issues^2–5^. In 2017, the combined cost of insufficient sleep across Canada, United States, United Kingdom, Germany and Japan was estimated to exceed $600 billion a year^6^. However, despite the global problem of sleep insufficiency and its serious public health implications, it is often inadequately recognised by individuals and health professionals alike^7,8^. This has motivated organizations such as the Centers for Disease Control and Prevention to advocate for increased surveillance of sleep problems, and heightened awareness of the health promoting effects of sleep^9^.

As ageing occurs there is often an ongoing deterioration in sleep quality and quantity that is associated with an increase in the prevalence of insomnia^10,11^. Both insufficient and excessive sleep have been associated with adverse effects among old adults including illness, respiratory issues, medication use, mental health issues (e.g., depression), and substance abuse^12–19^, with the lowest rates of morbidity and mortality among those sleeping 7–8 h per night^20^. Sex differences have also been reported whereby subjective sleep quality is typically worse in females than males^11,13^.

Concomitantly, with ageing, there is reduced social engagement, and increased loneliness, which predisposes older adults to negative health, well-being and mortality effects^21,22^. While still debated^22^, loneliness and social isolation are often regarded as inter-related but distinct concepts^23^. Here, loneliness is defined as an emotional state that arises when there is a perceived discrepancy between desired levels of social interaction, companionship, or emotional support, and that which is available to a person^24–26^. Whereas, social isolation is defined by having little or no social interaction with others^27^. So one can be socially isolated yet not lonely, and vice versa. The negative effects of reduced social engagement and loneliness have considerable research backing. Loneliness is associated with reduced physical health and activity^28–30^, and wellbeing^31^; and, increased mortality^32,33^, depression^34,35^, and alcohol consumption rates^36^. Social isolation, in particular, living alone exacerbates these problems^22,37,38^. However, the contribution of loneliness and social isolation to community living older adults sleep problems is neither well understood nor widely researched.

There exists only a small corpus of research demonstrating the relationship between loneliness, social isolation, and sleep quality among adults in general population studies^39,40^. This research suggests that loneliness results in decreased sleep quality^29,41–44^, often due to increased disruption rather than through substantive total sleep time or daytime sleepiness changes^29,45,46^. Conversely, living with another person has been associated with improved sleep quality^14,22^. The relationship between loneliness, social isolation, and sleep problems in older adults is likely to be complex, and mediated by a number of factors. Moreover, the relationship between loneliness, social isolation and sleep are likely to be age and sex dependent, requiring longitudinal sex-stratified approaches. Indeed, this is explicitly recognised in the call for further research that investigates how these associations may change as a function of sex and age^39^.

Since 2012 all adults seeking publicly-funded community care support in New Zealand have undergone a standardized comprehensive clinical assessment using the Home Care International Residential Assessment Instrument (interRAI-HC)^47^. This 236 item-based assessment tool covers multiple domains including loneliness, social isolation, and sleep problems, together with demographic, physical, mental and social questions. It is primarily designed and used to determine levels of need for health care services, develop care plans, and identify suitable support and service options for older individuals. Re-assessments are only undertaken if patient’s needs change, requiring additional support. Over 10% of all New Zealand adults aged ≥ 65 years have completed an interRAI-HC, with approximately 93% consenting to their data being used for planning and research purposes^47^.

Drawing on these interRAI-HC data, stratified by sex, the primary aims of this epidemiological investigation is to: (1) cross-sectionally describe the sleep problems and loneliness and social isolation profiles of community-dwelling older adults with complex needs; and, (2) to longitudinally evince these associations after controlling for socio-demographic and potentially confounding variables after employing apposite biostatistical methods.

## Methods

### Study design

Cross-sectional and longitudinal analysis of routinely collected data from a continuously recruited national cohort.

### Participants

Community dwelling women and men having an interRAI-HC assessment between 1 July 2012 and 31 May 2018, inclusive, aged ≥ 65 years, and who consented to their data being used for planning and research purposes. Those with sex not recorded as female/male or who were in hospital within 7 days of their assessment were excluded.

### Primary measures

The interRAI-HC assessment form, modified with permission for New Zealand, is used under license to the Ministry of Health (www.interrai.co.nz)^47^. Sleep problems were elicited by asking participants where they had: (1) difficulty falling asleep or staying asleep; waking up too early; restlessness; non-restful sleep (subsequently referred to as insufficient sleep); and (2) too much sleep (subsequently referred to as excessive sleep), defined within the interRAI-HC as an excessive amount that interferes with their normal functioning. Response options for both were: (0) not present; (1) present but not exhibited in last 3 days; (2) exhibited on 1 of last 3 days; (3) exhibited on 2 of last 3 days; and (4) exhibited daily in last 3 days. Participants were also asked about their fatigue, defined within the interRAI-HC as an inability to complete normal daily activities; with response options: (0) none; (1) minimal—diminished energy but completes normal day-to-day activities; (2) moderate—due to diminished energy, unable to finish normal day-to-day activities; (3) severe—due to diminished energy, unable to start some normal day-to-day activities; and (4) unable to commence any normal day-to-day activities—due to diminished energy. The variable *insufficient sleep* combined the first sleep (SP1) and fatigue variables, was rated as: none/minimal if (SP1 = 0) or (0 ≤ fatigue ≤ 1); moderate if (1 ≤ SP1 ≤ 2) and (2 ≤ fatigue ≤ 4); and severe if (3 ≤ SP1 ≤ 4) and (2 ≤ fatigue ≤ 4). The variable *excessive sleep* combined with fatigue variables, was similarly defined. As only a single item was used to measure sleep insufficiency and excessive sleep, respectively, reports of nocturnal and daytime sleep were combined with subjective ratings of fatigue to determine whether sleep was in fact problematic (i.e., likely to impact an individual’s daytime functioning); a diagnostic characteristic of sleep problems.

Loneliness was elicited by asking whether participants said or indicated that she/he feels lonely; with response options: (0) no; and (1) yes. Social isolation was defined by the living arrangements at the time of the assessment, and ascertained by asking participants who lived in their home; with response options: (1) alone; (2) with spouse/partner only; (3) with spouse/partner and other(s); (4) with child (not spouse/partner); (5) with parent(s) or guardian(s); (6) with siblings(s); (7) with other relatives; and, (8) with non-relative(s). The last seven categories were combined, i.e. (2–7), and relabelled lives with others. Because of the inter-relationship between loneliness and social isolation, rather than investigating main effects and interaction terms, these two variables were combined to form a single *loneliness/social isolation* variable, defined by: not lonely/lives with others; not lonely/lives alone; lonely/lives with others; and, lonely/lives alone.

### Socio-demographic and potential confounder measures

Age was derived by differencing interRAI-HC assessment from birth dates. Sex response options were: male, female, unknown, and indeterminate; responses to the last two options were set to missing. Participants were able to self-identify up to a maximum of three ethnic groups. Those with multiple ethnicity identifications were prioritised according to the Ministry of Health’s hierarchy protocol^48^, with Māori having priority coding, followed by Pacific, New Zealand European, and Other. Variables measuring marital status, cognitive impairment, pain, mood, smoking status, alcohol consumption, participation in activities of daily living (ADL), instrumental activities of daily living (IADL) capacity, urinary incontinence, medications, disease diagnoses, cardiac or pulmonary problems, psychiatric problems, gastrointestinal problems, aspiration problems, dyspnoea (shortness of breath), and self-rated health were included within the adjusted analyses.

### Procedures

The interRAI-HC procedure has been described in detail previously^47^. In brief, health practitioners refer individuals for an interRAI-HC assessment. Trained assessors conduct the assessment in clients’ own homes and produce individualized care-plans according to a standardized protocol. The interRAI-HC information is stored electronically and is National Health Index (NHI) number-linked, using encryption for data security. The NHI is a unique identifier that is assigned to every person who uses health and disability support services in New Zealand. Further information on interRAI-HC can be found at: www.interrai.co.nz.

### Statistical analysis

Reporting of analyses were informed by the REporting of studies Conducted using Observational Routinely-collected Data (RECORD) guidelines^49^. Participants’ repeated interRAI-HC assessments were deterministically matched using their unique encrypted NHI numbers. All analyses were stratified by sex. In accordance with the RECORD guidelines, the participant flow and descriptive summary statistics of participants’ socio-demographics were presented. Study aim (1) used participants’ first (baseline) assessments only. Here the sleep problem and loneliness/social isolation variables were descriptively reported. Next longitudinal analyses were undertaken on all available data (study aim 2), using random-effects ordered logistic regression models, grouped on participants, with robust Huber-White sandwich variance estimators. Treating age as a continuous variable, linear and quadratic changes in sleep problems with increasing age were initially investigated. Crude analyses then ensued, relating the loneliness/social isolation variable of interest and each of the potentially confounding variables separately to the insufficient sleep and excessive sleep variables, after account for the previously observed age patterns. In the spirit of Sun and colleagues^50^, all variables were then included within the adjusted complete case random-effects ordered logistic regression models regardless of their statistical significance. Crude odds ratios (ORs) and adjusted ORs, together with their associated 95% confidence intervals (CIs), were reported and Wald’s type III χ^2^ statistic used to determine a variable’s significance. All analyses were performed using Stata SE version 16.0 (StataCorp, College Station, TX, USA), and α = 0.05 defined significance.

### Ethics

Clearance for this study and its protocol was approved by the Ministry of Health’s Health and Disability Ethics Committee (14/STH/140). All methods were performed in accordance with that Ethics Committee’s relevant guidelines and regulations. The study only included those participants who provided written and informed consent to their data being used for planning and research purposes. The Ministry of Health does not release data to researchers for those who do not provide this consent. As this investigation is a secondary analysis of routine collected de-identified data, written and informed consent was not obtained for this specific study. The need for informed consent was waived by the Ministry of Health’s Health and Disability Ethics Committee for this study. The research databases released by the Ministry of Health contained participants’ encrypted NHI numbers, needed for matching, but has all personally identifying information removed. The encryption code is held by the Ministry of Health and no other.

## Results

### Sample description

The extracted interRAI-HC dataset contained 151,694 assessments from 102,178 participants. However, 117 assessments had been double entered, 7197 assessments were undertaken from 4949 adults aged < 65 years, sex was indeterminate for 2, unknown for 2 and missing for 47 participants, and 3905 assessments were conducted on participants who had been in hospital within the last 7 days, leaving an eligible database of 140,423 (87,245 from women; 62.1%) assessments from 95,045 (57,958 women, 61.0%) participants. Of these, 62,437 (65.7%) participants had one assessment; 23,221 (24.4%) had two; 6832 (7.2%) had three, 1860 (2.0%) had four; 577 (0.6%) had five; 104 (0.1%) had six, 13 (0.01%) had seven; and, 1 (< 0.01%) participant had eight interRAI-HC assessments. The median time between assessments was 1.60 years (Q_1_ = 0.98, Q_3_ = 2.63 years), with the largest assessment interval being 5.71 years. The average age at first assessment was 82.4 years (range 65–109 years) for women and 81.3 (range 65, 105 years) for men. Their demographic profiles are presented in Table 1. Most participants were aged 75–84 years and of New Zealand European ethnicity. However, women were more likely to be widowed (*p* < 0.001), older (*p* < 0.001) and be of Māori (*p* = 0.02) ethnicity than their male counterparts.

**Table 1:** Distribution of participant socio-demographics for 57,958 women and 37,087 men participants living at home at their first assessment.

	Women		Men	
	n	(%)	n	(%)
**Age (years)**				
65–74	9747	(16.8)	7516	(20.3)
75–84	23,329	(40.3)	15,788	(42.6)
85–94	22,655	(39.1)	12,938	(34.9)
95 +	2227	(3.8)	845	(2.3)
**Ethnicity**				
European	50,574	(87.3)	32,566	(87.8)
Māori	3625	(6.3)	2094	(5.6)
Pacific	1785	(3.1)	1150	(3.1)
Other	1974	(3.4)	1277	(3.4)
**Marital status**^a^				
Married/de facto	16,853	(29.1)	21,661	(58.5)
Widowed	34,668	(59.9)	9692	(26.2)
Divorced/separated	3911	(6.8)	3338	(9.0)
Never married	2043	(3.5)	2010	(5.4)
Other	448	(0.8)	358	(1.0)

^a^Missing values for 35 (0.06%) women and 28 (0.08%) men participants.

### Sleep problem profiles at baseline

At baseline, 50,769 (87.6%) women and 32,363 (87.3%) men reported having enough sleep, 2285 (3.9%) women and 1370 (3.7%) men had moderate sleep insufficiency, and 4901 (8.5%) women and 3353 (9.0%) men had severe sleep insufficiency; a difference that was significant between sexes (*p* = 0.02). In terms of excessive sleep, at baseline, 55,258 (95.3%) women and 34,269 (92.4%) men had no/minimal problems, 653 (1.1%) women and 614 (1.7%) men had moderate problems, and 2044 (3.5%) women and 2203 (5.9%) men had severe problems; with again significant difference between sexes (*p* < 0.001).

When comparing the two sleep measures at baseline, 49,024 (84.6%) women and 30,507 (82.3%) men reported no/minimal issues, while 527 (0.9%) women and 540 (1.5%) men were classified as having severe problems for both; see Table S1 in the supplementary materials. Association between these two variables was weak (Kendall's τ_b_ = 0.15 for women and 0.18 for men), although significant asymmetry was observed for both women (*p* < 0.001) and men (*p* < 0.001), with sleep classifications more likely to be poorer for sleep insufficiency compared to excessive sleep variables than vice versa.

### Loneliness/social isolation profiles at baseline

At baseline, 13,659 (23.6%) women and 7022 (18.9%) men reported being lonely (7 women and 6 men had missing values), while 31,205 (53.8%) women and 12,534 (33.8%) men reported living alone. When considered together, 22,404 (38.7%) women and 21,596 (58.2%) men were neither lonely nor living alone, 21,888 (37.8%) women and 8463 (22.8%) men were not lonely but living alone, 4342 (7.5%) women and 2952 (8.0%) men were lonely and living with others, and 9317 (16.1%) women and 4070 (11.0%) men were both lonely and living alone. A significant sex difference was observed (*p* < 0.001); among those who reported being lonely, more women were living alone than men.

### Relating loneliness/social isolation to sleep problems at baseline

Table 2 presents the cross-tabulations of the loneliness/social isolation and sleep problem variables at baseline, stratified by sex. Significant differences were observed in these crude results (all *p* < 0.001), with those who were lonely having higher observed rates of sleep insufficiency problems than those who were not lonely, whereas those living with others having higher rates of problems with excessive sleep than those living alone.

**Table 2:** Distribution of sleep problem and loneliness/social isolation characteristics for 57,958 women and 37,087 men living at home at their first assessment.

	Not enough sleep						Excessive sleep					
	None/minimal		Moderate		Severe		None/minimal		Moderate		Severe	
	n	(%)	n	(%)	n	(%)	n	(%)	n	(%)	n	(%)
**Women**^a^												
Not lonely/lives with others	19,680	(87.8)	846	(3.8)	1878	(8.4)	21,044	(93.9)	279	(1.2)	1081	(4.8)
Not lonely/lives alone	19,651	(89.8)	749	(3.4)	1486	(5.8)	21,190	(96.8)	183	(0.8)	513	(2.3)
Lonely/lives with others	3579	(82.4)	228	(5.3)	534	(12.3)	4069	(93.7)	70	(1.6)	202	(4.7)
Lonely/lives alone	7852	(84.3)	462	(5.0)	1003	(10.8)	8950	(96.1)	121	(1.3)	246	(2.6)
**Men**^b^												
Not lonely/lives with others	18,885	(87.5)	800	(3.7)	1910	(8.8)	19,625	(90.9)	392	(1.8)	1578	(7.3)
Not lonely/lives alone	7640	(90.3)	252	(3.0)	571	(6.7)	8107	(95.8)	101	(1.2)	255	(3.0)
Lonely/lives with others	2397	(81.2)	169	(5.7)	386	(13.1)	2659	(90.1)	62	(2.1)	231	(7.8)
Lonely/lives alone	3436	(84.4)	149	(3.7)	485	(11.9)	3873	(95.2)	59	(1.4)	138	(3.4)

^a^Missing sleep values for 10 (0.02%) women.

^b^Missing sleep values for 7 (0.02%) men.

### Sleep problem profiles over time

**Figure 1:**
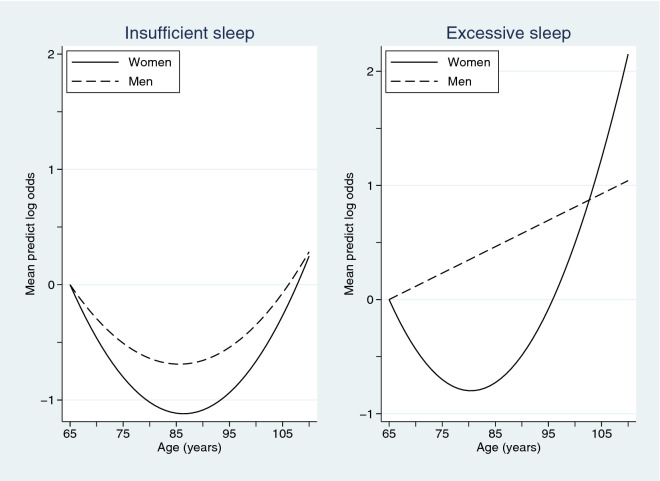
Mean predicted log odds of the two sleep variables over age, partitioned by sex, using age 65 years as the reference level.

Applying random-effects ordered logistic regression models stratified by sex, a significant quadratic mean change in sleep insufficiency problems was observed for women (*p* < 0.001) and men (*p* < 0.001) as they aged; see Fig. 1. However, the proportion of participant’s attributable variance was 72.9% for women and 75.2% for men, suggesting that individual variation accounts for most of the regression model variance.

Re-applying the random-effects ordered logistic regression models to the excessive sleep variable, a significant quadratic change was again observed for women (*p* < 0.001) but not men (*p* = 0.10) as they aged. However, for men, a significant linear increase with age (*p* < 0.001) was identified; see Fig. 1. The proportion of participant’s attributable variance in these models was 74.4% for women and 78.6% for men, again suggesting that individual variation explains most of the model variance.

### Relating loneliness/social isolation to sleep problems over time: crude analysis

Applying the random-effects ordered logistic regression models to the full dataset, stratified by sex and adjusted for the identified age patterns, significant associations were found between the loneliness/social isolation and the two sleep problem variables (all *p* < 0.001). Table 3 presents the crude OR and associated 95% confidence intervals. For reported sleep insufficiency among both women and men, those not lonely and living alone had the smallest likelihood, while those lonely and living with others had the highest likelihood. Those who were lonely and living with others had significantly higher estimated odds of sleep insufficiency than those who were lonely and living alone for both women (*p* = 0.03) and men (*p* < 0.001). For excessive sleep, those not lonely and living with others had estimated odds no different from those who were lonely and living with others (*p* = 0.68 for women and *p* = 0.08 for men). Those who were living alone had significantly lower odds than those who were not lonely for women (*p* < 0.001) but not for men (*p* = 0.07).

**Table 3:** Crude and adjusted ORs, together with their associated 95% CI, derived from random-effects ordered logistic regression models relating loneliness/social isolation characteristics to the sleep problem variables for 57,958 women and 37,087 men living at home across all interRAI-HC assessments.

	Not enough sleep				Excessive sleep			
	Crude^a^		Adjusted^b^		Crude^c^		Adjusted^b^	
	OR	(95% CI)	OR	(95% CI)	OR	(95% CI)	OR	(95% CI)
**Women**								
Not lonely/lives with others	1	(reference)	1	(reference)	1	(reference)	1	(reference)
Not lonely/lives alone	0.83	(0.76, 0.91)	1.09	(0.98, 1.22)	0.29	(0.25, 0.34)	0.72	(0.60, 0.86)
Lonely/lives with others	2.14	(1.88, 2.45)	1.25	(1.08, 1.44)	1.04	(0.86, 1.26)	0.81	(0.65, 1.00)
Lonely/lives alone	1.83	(1.65, 2.03)	1.46	(1.28, 1.66)	0.48	(0.41, 0.57)	0.76	(0.62, 0.94)
**Men**								
Not lonely/lives with others	1	(reference)	1	(reference)	1	(reference)	1	(reference)
Not lonely/lives alone	0.62	(0.55, 0.71)	1.05	(0.88, 1.24)	0.23	(0.19, 0.29)	0.63	(0.50, 0.80)
Lonely/lives with others	2.23	(1.90, 2.63)	1.40	(1.18, 1.67)	1.19	(0.98, 1.44)	0.87	(0.69, 1.10)
Lonely/lives alone	1.51	(1.31, 1.96)	1.61	(1.33, 1.96)	0.30	(0.24, 0.38)	0.59	(0.45, 0.79)

^a^Adjusted for age and age^2^.

^b^Adjusted the age pattern used in the crude analysis together with loneliness/social isolation, ethnicity, marital status, cognitive impairment, pain, mood, smoking status, alcohol consumption, participation in activities of daily living (ADL), instrumental activities of daily living (IADL) capacity, urinary incontinence, medications, disease diagnoses, cardiac or pulmonary problems, psychiatric problems, gastrointestinal problems, aspiration problems, dyspnoea (shortness of breath), and self-rated health; 82 (0.09%) interRAI-HC assessments were omitted due to missing values.

^c^adjusted for age and age^2^ for women and age only for men.

### Relating loneliness/social isolation to sleep problems over time: adjusted analysis

The adjusted ORs derived from the complete case random effects ordinal logistic regression models of insufficient sleep and excessive sleep, stratified by sex, appear in Table 3 and Tables S2–S5 within the supplementary materials. For the insufficient sleep variable, there continued to be a significant association with loneliness/social isolation after confounder adjustment for women (*p* < 0.001) and men (*p* < 0.001). Compared to those not lonely and living with others, both women and men who were lonely and living alone had the highest estimated odds for not having enough sleep of 1.46 (95% CI 1.28, 1.66) and 1.61 (95% CI 1.33, 1.96), respectively. However, there was no significant difference in the estimated odds between lonely women or men who were living alone or living with others (*p* = 0.06 and *p* = 0.24, respectively). Despite the large number of significant confounders identified, the proportion of variance attributable to individual women in the random effects model was 69.5% while it was 71.3% in the model for men.

For the excessive sleep variable, a significant association with loneliness/social isolation was also maintained in the adjusted analysis for women (*p* = 0.002) and men (*p* < 0.001). However, unlike the sleep insufficient variable, those who were living alone (regardless of whether they were lonely on not) had significantly lower estimated odds than those who were living with others—for both women and men; see Table 3. And there was no significant difference in the estimated odds between women or men who were living alone and whether they were lonely or not (*p* = 0.54 and *p* = 0.65, respectively). The proportion of variance attributable to individuals within this random effects model was 77.6% for women and 78.8% for men.

## Discussion

This national population study investigated the prevalence of sleep insufficiency, excessive daytime sleepiness, and self-reported loneliness and social isolation among a community cohort of older people with complex health needs. Data were stratified by sex, and crude and adjusted ORs were calculated to identify age trends in sleep-related problems and control for confounding demographic, adaptive functioning, physical and mental health variables. Empirical findings revealed that many older adults experience problems with insufficient sleep, which significantly changed with age. Relatively fewer also experience problems with excessive sleep that is reported to interfere with their daytime functioning. Interestingly, there were relatively few instances in which people reported both types of sleep problems suggesting that excessive daytime sleep was not a result of nocturnal sleep deprivation and vice versa. Instead, it is possible that problems with insufficient and excessive sleep are the result of different underlying mechanisms (e.g., physical health problem or mood disorder).

For both women and men there was a reduction in reported problems with sleep insufficiency as they aged (i.e., between the period of 65–85 years). However, there was an increase in sleep insufficiency problems among those over 85 years of age. A similar trend was identified for women in regard to excessive sleep whereby there was a decline in reported problems with daytime sleep as participants aged, until approximately 80 years of age, at which point there was an increase in problems with excessive sleep. By contrast, for men there was a linear pathway whereby more men reported problems with excessive sleep as they aged. Interestingly, problems with both severe sleep insufficiency and excessive sleep were more frequent among men than women. With the exception of excessive sleep problems among men, these age-related changes and sex differences were largely unexpected, and are in contrast to those reported in existing research^10,11^. However, these findings could be attributed to a number of possibilities inherent within the data. Firstly, it is noteworthy that the effect sizes and ORs across a number of confounding variables such as pain, mood, medication use, and dyspnoea, were large (see Table S2). This suggests a number of potential confounding factors were associated with sleep problems in this population. Secondly, due to the vulnerability of the population that was sampled, it is likely that the respondents had a number of co-occurring physical and mental health problems, many of which increase with age^51^. As such, the determination of whether sleep was problematic may be relative to the number of co-existing problems, and these are likely to be greater among the older age range. It is also important to note that the proportion of attributable variance was high across sleep categories. This suggests that individual trajectories were variable. As a result, the observed mean trends should be interpreted with caution. Finally, given the study’s age limits, it is possible that the sleep problems reported here do reflect a deterioration in sleep compared to those under 65 years of age.

Almost one quarter of women and one fifth of men reported feeling lonely. This feeling of loneliness was more common among both women and men when living alone. Findings suggest that those who were lonely, regardless of whether living alone or with others, were more likely to report sleep insufficiency problems than those who were not lonely. Moreover, both women and men who were living alone and lonely had the greatest likelihood of problems with insufficient sleep. By contrast, both women and men who were living with others who were not lonely had the least problems with sleep insufficiency. While there is a paucity of related research, these findings are consistent with previous studies which suggest that it is in fact emotional loneliness, as opposed to social isolation, which is primarily associated with sleep problems in older people^42,43^. However, as indicated by Yu et al., both social isolation and loneliness may have a distinct role in sleep problems^44^. It is possible that the effect of social isolation on sleep problems may be more likely to persist long-term in comparison to self-reported loneliness^44^, or as the current findings suggest, social isolation may exacerbate sleep problems in those who are lonely.

An unanticipated finding was that, for both women and men, those living with others were more likely to report problems with excessive sleep than those living alone, regardless of whether they were lonely. There is a dearth of research that has examined the relationship between loneliness/social isolation and excessive sleep and thus it is difficult to contextualize this finding within the current literature. While we have endeavoured to examine an extensive list of confounders, it is plausible that there is a secondary mechanism that may explain problems with excessive sleep. Residual confounding factors may explain both excessive sleepiness and also why the person is living with others (i.e., for personal cares). This raises an important consideration for future research.

The findings of this study should be considered in light of its limitations. The first relates to the sample of participants. The interRAI-HC data was collected from over 10% of community dwelling older people located throughout New Zealand^47^. This resulted in an exceptionally large data set that afforded opportunities for a number of complex analyses. However, the instrument itself is used with older people who may require access to publically funded services or aged residential care. While not all of those assessed met necessary thresholds for additional support, the data obtained may be more representative of those with complex needs that necessitate secondary care as opposed to a community-based population, thus limiting the external validity of the study findings. Additionally, most of the participants (65.7%) had only one assessment available for the longitudinal analyses, yet temporal patterns could only be explored for those participants with repeated assessments. The implicit assumption is that those without repeated interRAI-HC assessments behave in a similar way as those who do, after adjusting for confounders. There are various reasons why some older adults might have a restricted number of assessments (such as death or moving to an aged residential care facility—whereby interRAI-HC assessments are no longer applicable). Moreover, many of the repeated assessments were routine or return, without a significant change in health status. Nonetheless, if those with one assessment have patterns of loneliness, social isolation and sleep problems that are systematically different from older adults with multiple assessments, then bias may affect the reported temporal findings.

A second limitation relates to the measure itself. The interRAI-HC only provides a rudimentary measure of sleep problems that is dependent on subjective self-reported sleep over the past three nights. It is unclear whether the sleep data collected accurately captures the type or severity of sleep problems experienced by older people. Furthermore, only a single item was used within the interRAI-HC to assess sleep insufficiency and excessive sleep respectively. As only a single item was used it is not possible to detect whether the self-reported sleep problems meet the diagnostic threshold for insomnia, nor is it possible to differentiate between problems with sleep quality and quantity. To partially address this issue, the items relating to sleep were combined with daytime fatigue to ensure that sleep problems were sufficient to impact daytime functioning. While it could be argued that daytime fatigue should be treated as a separate variable, there are a number of factors beyond sleep deprivation or disruption that may result in daytime fatigue thus, it was not appropriate to explore fatigue as a standalone variable. Additionally, the DSM-V criteria for insomnia^52^, along with psychometric measures of adult sleep quality and quantity^12^ include a dimension that assesses whether the sleep problem results in daytime functional impairment. Daytime fatigue, assessed in this study as an inability to complete normal daytime activities, provides this measure of impact. In future research, to gather a comprehensive picture of the type and severity of sleep problems, it may be necessary to conduct objective sleep measurement (e.g., polysomnography), gather data over a sustained period of time, and/or use multiple measures to permit the triangulation of sleep data^13^. This is particularly important given the risk of cognitive decline in the aging population, and the levels of recall necessary for many subjective self-report measures. The discrepancy between objective and subjective sleep measures reported in the literature adds credence to this argument^53^. Given that the literature also suggests that sleep quality, as opposed to quantity, is associated with loneliness, future research should also endeavour to use measures capable of this differentiation. Another potential limitation of this study is that a binary variable was used to assess perceptions of loneliness. This fails to capture some of the nuanced experience of feeling lonely, and does not distinguish between acute and chronic experiences of loneliness. Future research should consider incorporating more sophisticated measures of both loneliness and sleep. Finally, it is important for future research to assess age-related changes in homeostatic sleep pressure and circadian physiology, and how this may interact with social and environmental factors^54,55^.

## Conclusions

This is the first study to examine the association between perceived loneliness and social isolation and problems with both insufficient and excessive sleep, among older people, and how these relationships may change as a function of age. This study is also unique in that it controlled for an extensive number of potential confounders that may have impacted upon the association between loneliness/social isolation and sleep, not previously considered in research. The study findings have a number of important implications for service delivery and supports. Given the implications of both loneliness and sleep problems for mortality and morbidity in older people, it is essential that we carefully assess these problems, regardless of an individuals’ living situation. Study findings also reflect the importance of assessing problems with excessive sleep particularly, as they may impair daytime functioning and limit access to social opportunities. Beyond assessment, it is also important that these findings are materialised to inform the provision of supports and services that may prevent or remediate loneliness in older people. In particular, it can be important to address loneliness as it may impact on the salubrity of restorative behaviours, such as sleep. Finally, it is possible that both sleep problems and loneliness may be symptomatic of other underlying or residual problems that require careful assessment.

## Supplementary Information

Supplementary Information.

## Data Availability

The datasets used for statistical analysis are held by New Zealand’s Ministry of Health. Application to use these data must be made through this Ministry.
