# Supplementary material for: Loneliness and social isolation is associated with sleep problems among older community dwelling women and men with complex needs
Source: Sci Rep. 2021 Mar 1;11:4877. doi: 10.1038/s41598-021-83778-w (PMC7921547; doi:10.1038/s41598-021-83778-w)
Supplement: Supplementary file 1 — Supplementary Information. [file 41598_2021_83778_MOESM1_ESM.docx]

**Supplementary materials for:**

**Loneliness and social isolation is associated with sleep problems among older community dwelling women and men with complex needs**

Laurie McLay*^1^, Hamish A. Jamieson^2,3^, Karyn G. France^1^, Philip J. Schluter^1,4^

1. University of Canterbury – Te Whare Wānanga o Waitaha, School of Health Sciences, Christchurch, New Zealand
2. University of Otago, Department of Medicine, Christchurch, New Zealand
3. Canterbury District Health Board, Older Person’s Health Christchurch, New Zealand
4. The University of Queensland, School of Clinical Medicine, Primary Care Clinical Unit, Brisbane, Australia

**Corresponding author*: Associate Professor Laurie McLay, University of Canterbury – Te Whare Wānanga o Waitaha, School of Health Sciences, Private Bag 4800, Christchurch 8140, New Zealand. Email: laurie.mclay@canterbury.ac.nz

**Table S1.** Cross-tabulations of the two sleep problem variables assessed at baseline (first assessment), stratified by sex.

|  |  | Too much sleep | | | | | |
| --- | --- | --- | --- | --- | --- | --- | --- |
|  | | None/minimal | | Moderate | | Severe | |
| Too little sleep | | n | (%) | n | (%) | n | (%) |
| *Women* | |  |  |  |  |  |  |
|  | None/minimal | 49,024 | (84.6) | 365 | (0.6) | 1,380 | (2.4) |
|  | Moderate | 1,997 | (3.4) | 151 | (0.3) | 137 | (0.2) |
|  | Severe | 4,237 | (7.3) | 137 | (0.2) | 527 | (0.9) |
| *Men* | |  |  |  |  |  |  |
|  | None/minimal | 30,507 | (82.3) | 327 | (0.9) | 1,529 | (4.1) |
|  | Moderate | 1,098 | (3.0) | 138 | (0.4) | 134 | (0.4) |
|  | Severe | 2,664 | (7.2) | 149 | (0.4) | 540 | (1.5) |

**Table S2**. Distribution of factors by the not enough sleep variable for women at first assessment, together with their estimated crude and adjusted odds ratios (ORs) and associated 95% confidence intervals (CIs) in the complete case random effects ordinal logistic regression over all assessments.

|  |  | Not enough sleep | | | | | |  | |  | |
| --- | --- | --- | --- | --- | --- | --- | --- | --- | --- | --- | --- |
|  |  | None/minimal | | Moderate | | Severe | | Crude* | | Adjusted† | |
| **Women** | | n | (%) | n | (%) | n | (%) | OR | (95% CI) | OR | (95% CI) |
| *Loneliness/social isolation* | |  |  |  |  |  |  |  |  |  |  |
|  | Not lonely/lives with others | 19,680 | (87.8) | 846 | (3.8) | 1,878 | (8.4) | 1 | (reference) | 1 | (reference) |
|  | Not lonely/lives alone | 19,651 | (89.8) | 749 | (3.4) | 1,486 | (6.8) | 0.83 | (0.76, 0.91) | 1.09 | (0.98, 1.22) |
|  | Lonely/lives with others | 3,579 | (82.4) | 228 | (5.3) | 534 | (12.3) | 2.14 | (1.88, 2.45) | 1.25 | (1.08, 1.44) |
|  | Lonely/lives alone | 7,852 | (84.3) | 462 | (5.0) | 1,003 | (10.8) | 1.83 | (1.65, 2.03) | 1.46 | (1.28, 1.66) |
| *Ethnicity* | |  |  |  |  |  |  |  |  |  |  |
|  | European | 44,309 | (87.6) | 2,018 | (4.0) | 4,244 | (8.4) | 1 | (reference) | 1 | (reference) |
|  | Māori | 3,113 | (85.9) | 142 | (3.9) | 370 | (10.2) | 1.12 | (0.95, 1.31) | 1.09 | (0.92, 1.29) |
|  | Pacific | 1,639 | (91.8) | 42 | (2.4) | 104 | (5.8) | 0.40 | (0.31, 0.52) | 0.52 | (0.40, 0.68) |
|  | Other | 1,708 | (86.5) | 83 | (4.2) | 183 | (9.3) | 1.02 | (0.82, 1.26) | 0.93 | (0.75, 1.17) |
| *Marital status* | |  |  |  |  |  |  |  |  |  |  |
|  | Married/de facto | 14,495 | (86.0) | 745 | (4.4) | 1,613 | (9.6) | 1 | (reference) | 1 | (reference) |
|  | Widowed | 30,714 | (88.6) | 1,270 | (3.7) | 2,681 | (7.7) | 0.83 | (0.76, 0.91) | 0.71 | (0.64, 0.80) |
|  | Divorce/separated | 3,305 | (84.5) | 179 | (4.6) | 427 | (10.9) | 1.11 | (0.94, 1.30) | 0.79 | (0.66, 0.95) |
|  | Never married | 1,835 | (89.8) | 66 | (3.2) | 142 | (7.0) | 0.50 | (0.40, 0.64) | 0.61 | (0.47, 0.79) |
|  | Other | 391 | (87.3) | 22 | (4.9) | 35 | (7.8) | 0.68 | (0.43, 1.06) | 0.68 | (0.42, 1.11) |
| *Cognitive impairment* | |  |  |  |  |  |  |  |  |  |  |
|  | Intact | 18,600 | (86.0) | 933 | (4.3) | 2,098 | (9.7) | 1 | (reference) | 1 | (reference) |
|  | Borderline | 19,780 | (87.6) | 921 | (4.1) | 1,890 | (8.4) | 1.02 | (0.94, 1.11) | 0.87 | (0.79, 0.95) |
|  | Moderately impaired | 10,029 | (89.9) | 377 | (3.4) | 751 | (6.7) | 0.85 | (0.77, 0.94) | 0.68 | (0.60, 0.77) |
|  | Severe + | 2,352 | (91.6) | 54 | (2.1) | 162 | (6.3) | 0.77 | (0.64, 0.92) | 0.61 | (0.49, 0.77) |
| *Pain* | |  |  |  |  |  |  |  |  |  |  |
|  | No pain | 19,338 | (92.5) | 550 | (2.6) | 1,008 | (4.8) | 1 | (reference) | 1 | (reference) |
|  | Less than daily pain | 11,241 | (89.1) | 524 | (4.2) | 846 | (6.7) | 2.02 | (1.82, 2.23) | 1.46 | (1.31, 1.62) |
|  | Daily pain, not severe | 13,656 | (85.4) | 720 | (4.5) | 1,623 | (10.1) | 3.53 | (3.21, 3.89) | 2.13 | (1.92, 2.37) |
|  | Daily severe pain | 6,534 | (77.3) | 491 | (5.8) | 1,424 | (16.9) | 8.81 | (7.86, 9.88) | 3.71 | (3.27, 4.19) |
| *Mood* | |  |  |  |  |  |  |  |  |  |  |
|  | Low risk | 31,802 | (90.9) | 1,080 | (3.1) | 2,093 | (6.0) | 1 | (reference) | 1 | (reference) |
|  | Medium risk | 11,989 | (85.3) | 683 | (4.9) | 1,377 | (9.8) | 2.51 | (2.31, 2.73) | 1.71 | (1.56, 1.87) |
|  | High risk | 6,976 | (78.1) | 522 | (5.8) | 1,431 | (16.0) | 6.29 | (5.67, 6.97) | 2.95 | (2.65, 3.29) |
| *Smoking status* | |  |  |  |  |  |  |  |  |  |  |
|  | Non-smoker | 48,283 | (87.8) | 2,155 | (3.9) | 4,579 | (8.3) | 1 | (reference) | 1 | (reference) |
|  | Smoker | 2,486 | (84.6) | 130 | (4.4) | 322 | (11.0) | 1.23 | (1.04, 1.46) | 0.95 | (0.79, 1.14) |
| *Alcohol consumption* | |  |  |  |  |  |  |  |  |  |  |
|  | None | 41,634 | (87.4) | 1,869 | (3.9) | 4,124 | (8.7) | 1 | (reference) | 1 | (reference) |
|  | 1 | 6,648 | (88.0) | 328 | (4.3) | 578 | (7.7) | 0.84 | (0.76, 0.94) | 1.02 | (0.90, 1.14) |
|  | ≥2 | 2,487 | (89.7) | 88 | (3.2) | 199 | (7.2) | 0.68 | (0.57, 0.81) | 0.86 | (0.71, 1.05) |
| *ADL* | |  |  |  |  |  |  |  |  |  |  |
|  | Independent | 34,943 | (88.3) | 1,480 | (3.7) | 3,165 | (8.0) | 1 | (reference) | 1 | (reference) |
|  | Supervision | 5,826 | (88.7) | 260 | (4.0) | 481 | (7.3) | 1.23 | (1.10, 1.37) | 1.04 | (0.92, 1.17) |
|  | Limited | 4,588 | (84.2) | 284 | (5.2) | 577 | (10.6) | 2.26 | (2.02, 2.53) | 1.22 | (1.08, 1.38) |
|  | Extensive | 2,809 | (86.7) | 111 | (3.4) | 319 | (9.8) | 1.95 | (1.69, 2.25) | 1.07 | (0.91, 1.25) |
|  | Maximal | 1,032 | (81.6) | 71 | (5.6) | 161 | (12.7) | 2.90 | (2.36, 3.56) | 1.23 | (0.99, 1.54) |
|  | Dependent + | 1,568 | (85.0) | 79 | (4.3) | 198 | (10.7) | 2.29 | (1.89, 2.77) | 1.16 | (0.93, 1.43) |
| *IADL capacity* | |  |  |  |  |  |  |  |  |  |  |
|  | 0-14 | 13,884 | (90.2) | 478 | (3.1) | 1,028 | (6.7) | 1 | (reference) | 1 | (reference) |
|  | 15-24 | 12,904 | (87.3) | 624 | (4.2) | 1,249 | (8.5) | 1.93 | (1.73, 2.15) | 1.36 | (1.21, 1.52) |
|  | 25-34 | 12,258 | (85.7) | 620 | (4.3) | 1,421 | (9.9) | 2.98 | (2.66, 3.35) | 1.69 | (1.50, 1.91) |
|  | 35-48 | 11,720 | (86.9) | 563 | (4.2) | 1,203 | (8.9) | 3.14 | (2.78, 3.55) | 1.72 | (1.48, 2.00) |
| *Urinary incontinence* | |  |  |  |  |  |  |  |  |  |  |
|  | Continent | 30,289 | (89.3) | 1,196 | (3.5) | 2,436 | (7.2) | 1 | (reference) | 1 | (reference) |
|  | Occasionally incontinent | 10,067 | (85.6) | 554 | (4.7) | 1,146 | (9.7) | 1.90 | (1.73, 2.08) | 1.23 | (1.12, 1.35) |
|  | Frequently incontinent | 9,853 | (85.1) | 490 | (4.2) | 1,234 | (10.7) | 2.44 | (2.22, 2.68) | 1.32 | (1.20, 1.46) |
|  | Otherwise | 560 | (81.2) | 45 | (6.5) | 85 | (12.3) | 3.11 | (2.32, 4.16) | 1.41 | (1.04, 1.91) |
| *Medication CAP* | |  |  |  |  |  |  |  |  |  |  |
|  | No | 43,969 | (89.8) | 1,606 | (3.3) | 3,396 | (6.9) | 1 | (reference) | 1 | (reference) |
|  | Yes | 6,797 | (75.7) | 679 | (7.6) | 1,505 | (16.8) | 5.29 | (4.85, 5.78) | 1.46 | (1.33, 1.60) |
| *Number of disease diagnoses* | |  |  |  |  |  |  |  |  |  |  |
|  | 0 | 10,071 | (90.9) | 311 | (2.8) | 695 | (6.3) | 1 | (reference) | 1 | (reference) |
|  | 1 | 16,679 | (89.2) | 647 | (3.5) | 1,372 | (7.3) | 1.45 | (1.24, 1.39) | 1.11 | (0.99, 1.26) |
|  | 2 | 13,176 | (87.1) | 617 | (4.1) | 1,331 | (8.8) | 2.12 | (1.87, 2.40) | 1.20 | (1.06, 1.36) |
|  | 3 | 6,914 | (84.4) | 409 | (5.0) | 872 | (10.6) | 3.16 | (2.75, 3.62) | 1.31 | (1.14, 1.50) |
|  | 4 | 2,757 | (81.4) | 218 | (6.4) | 414 | (12.2) | 4.97 | (4.19, 5.90) | 1.60 | (1.34, 1.90) |
|  | ≥5 | 1,172 | (79.6) | 83 | (5.6) | 217 | (14.7) | 6.81 | (5.43, 8.52) | 1.57 | (1.25, 1.98) |
| *Chest pain* | |  |  |  |  |  |  |  |  |  |  |
|  | Not present | 45,678 | (88.9) | 1,803 | (3.5) | 3,923 | (7.6) | 1 | (reference) | 1 | (reference) |
|  | Present | 5,091 | (77.7) | 482 | (7.4) | 978 | (14.9) | 3.81 | (3.43, 4.23) | 1.40 | (1.26, 1.56) |
| *Difficulty clearing airway secretions* | | |  |  |  |  |  |  |  |  |  |
|  | Not present | 47,605 | (88.7) | 1,958 | (3.6) | 4,107 | (7.7) | 1 | (reference) | 1 | (reference) |
|  | Present | 3,164 | (73.8) | 327 | (7.6) | 794 | (18.5) | 5.61 | (4.93, 6.37) | 1.87 | (1.64, 2.13) |
| *Abnormal thought process* | |  |  |  |  |  |  |  |  |  |  |
|  | Not present | 49,296 | (87.6) | 2,219 | (3.9) | 4,737 | (8.4) | 1 | (reference) | 1 | (reference) |
|  | Present | 1,473 | (86.5) | 66 | (3.9) | 164 | (9.6) | 1.56 | (1.27, 1.90) | 1.32 | (1.06, 1.65) |
| *Delusions* | |  |  |  |  |  |  |  |  |  |  |
|  | Not present | 48,984 | (87.5) | 2,206 | (3.9) | 4,760 | (8.5) | 1 | (reference) | 1 | (reference) |
|  | Present | 1,785 | (89.0) | 79 | (3.9) | 141 | (7.0) | 1.01 | (0.84, 1.22) | 0.84 | (0.67, 1.04) |
| *Hallucinations* | |  |  |  |  |  |  |  |  |  |  |
|  | Not present | 48,761 | (87.8) | 2,162 | (3.9) | 4,626 | (8.3) | 1 | (reference) | 1 | (reference) |
|  | Present | 2,008 | (83.5) | 123 | (5.1) | 275 | (11.4) | 2.12 | (1.81, 2.49) | 1.65 | (1.39, 1.97) |
| *Acid reflux* | |  |  |  |  |  |  |  |  |  |  |
|  | Not present | 42,504 | (89.4) | 1,535 | (3.2) | 3,504 | (7.4) | 1 | (reference) | 1 | (reference) |
|  | Present | 8,265 | (79.4) | 750 | (7.2) | 1,397 | (13.4) | 3.65 | (3.34, 4.00) | 1.79 | (1.63, 1.97) |
| *Constipation* | |  |  |  |  |  |  |  |  |  |  |
|  | Not present | 40,611 | (89.5) | 1,453 | (3.2) | 3,317 | (7.3) | 1 | (reference) | 1 | (reference) |
|  | Present | 10,158 | (80.8) | 832 | (6.6) | 1,584 | (12.6) | 3.51 | (3.22, 3.82) | 1.72 | (1.58, 1.88) |
| *Diarrhoea* | |  |  |  |  |  |  |  |  |  |  |
|  | Not present | 47,056 | (88.4) | 1,946 | (3.7) | 4,212 | (7.9) | 1 | (reference) | 1 | (reference) |
|  | Present | 3,713 | (78.3) | 339 | (7.2) | 689 | (14.5) | 3.80 | (3.37, 4.27) | 1.83 | (1.62, 2.07) |
| *Vomiting* | |  |  |  |  |  |  |  |  |  |  |
|  | Not present | 49,762 | (88.0) | 2,165 | (3.8) | 4,647 | (8.2) | 1 | (reference) | 1 | (reference) |
|  | Present | 1,007 | (72.9) | 120 | (8.7) | 254 | (18.4) | 4.24 | (3.47, 5.18) | 1.37 | (1.11, 1.69) |
| *Aspiration problems* | |  |  |  |  |  |  |  |  |  |  |
|  | Not present | 50,429 | (87.7) | 2,255 | (3.9) | 4,828 | (8.4) | 1 | (reference) | 1 | (reference) |
|  | Present | 340 | (76.7) | 30 | (6.8) | 73 | (16.5) | 3.76 | (2.56, 5.53) | 1.28 | (0.86, 1.89) |
| *Dyspnoea (shortness of breath)* | |  |  |  |  |  |  |  |  |  |  |
|  | Absent | 28,570 | (92.2) | 735 | (2.4) | 1,673 | (5.4) | 1 | (reference) | 1 | (reference) |
|  | With moderate activities | 12,004 | (87.8) | 565 | (4.1) | 1,102 | (8.1) | 2.25 | (2.04, 2.47) | 1.41 | (1.27, 1.56) |
|  | With day-to-day activities | 7,699 | (79.2) | 657 | (6.8) | 1,362 | (14.0) | 7.57 | (6.79, 8.44) | 3.36 | (3.00, 3.77) |
|  | Present at rest | 2,496 | (69.6) | 328 | (9.1) | 764 | (21.3) | 17.4 | (15.0, 20.3) | 5.45 | (4.67, 6.37) |
| *Self-rated health* | |  |  |  |  |  |  |  |  |  |  |
|  | Excellent | 1,919 | (96.8) | 25 | (1.3) | 39 | (2.0) | 1 | (reference) | 1 | (reference) |
|  | Good | 24,214 | (93.5) | 583 | (2.3) | 1,096 | (4.2) | 2.76 | (2.03, 3.77) | 2.04 | (1.48, 2.80) |
|  | Fair | 16,832 | (84.2) | 1,055 | (5.3) | 2,104 | (10.5) | 12.9 | (9.47, 17.7) | 5.38 | (3.90, 7.42) |
|  | Poor | 4,159 | (70.6) | 471 | (8.0) | 1,260 | (21.4) | 48.2 | (34.8, 66.7) | 9.44 | (6.75, 13.2) |
|  | No response | 3,645 | (86.8) | 151 | (3.6) | 402 | (9.6) | 10.1 | (7.29, 14.1) | 5.09 | (3.60, 7.19) |

Note: *Adjusted for age and age^2^; †Adjusted for age, age^2^, loneliness/social isolation, ethnic identification, marital status, cognitive impairment, pain, mood CAP, smoking status, alcohol consumption, ADL, IADL capacity, urinary incontinence, medication CAP, number of disease diagnoses, chest pain, difficulty clearing airway secretions, abnormal thought process, delusions, hallucinations, acid reflux, constipation, diarrhoea, vomiting, aspiration problems, dyspnoea, and self-rated health. In the adjusted analyses, 82 (0.09%) interRAI-HC assessments were omitted due to missing values.

**Table S3**. Distribution of factors by the not enough sleep variable for men at first assessment, together with their estimated crude and adjusted odds ratios (ORs) and associated 95% confidence intervals (CIs) in the complete case random effects ordinal logistic regression over all assessments.

|  |  | Not enough sleep | | | | | |  | |  | |
| --- | --- | --- | --- | --- | --- | --- | --- | --- | --- | --- | --- |
|  |  | None/minimal | | Moderate | | Severe | | Crude* | | Adjusted† | |
| **Men** | | n | (%) | n | (%) | n | (%) | OR | (95% CI) | OR | (95% CI) |
| *Loneliness/social isolation* | |  |  |  |  |  |  |  |  |  |  |
|  | Not lonely/lives with others | 18,885 | (87.5) | 800 | (3.7) | 1,910 | (8.8) | 1 | (reference) | 1 | (reference) |
|  | Not lonely/lives alone | 7,640 | (90.3) | 252 | (3.0) | 571 | (6.7) | 0.62 | (0.55, 0.71) | 1.05 | (0.88, 1.24) |
|  | Lonely/lives with others | 2,397 | (81.2) | 169 | (5.7) | 386 | (13.1) | 2.23 | (1.90, 2.63) | 1.40 | (1.18, 1.67) |
|  | Lonely/lives alone | 3,436 | (84.4) | 149 | (3.7) | 485 | (11.9) | 1.51 | (1.31, 1.76) | 1.61 | (1.33, 1.96) |
| *Ethnicity* | |  |  |  |  |  |  |  |  |  |  |
|  | European | 28,400 | (87.2) | 1,217 | (3.7) | 2,948 | (9.1) | 1 | (reference) | 1 | (reference) |
|  | Māori | 1,799 | (85.9) | 75 | (3.6) | 220 | (10.5) | 1.10 | (0.88, 1.36) | 1.02 | (0.81, 1.28) |
|  | Pacific | 1,030 | (89.6) | 31 | (2.7) | 89 | (7.7) | 0.59 | (0.44, 0.81) | 0.54 | (0.40, 0.75) |
|  | Other | 1,134 | (88.8) | 47 | (3.7) | 96 | (7.5) | 0.68 | (0.51, 0.91) | 0.59 | (0.43, 0.79) |
| *Marital status* | |  |  |  |  |  |  |  |  |  |  |
|  | Married/de facto | 18,693 | (86.3) | 901 | (4.2) | 2,066 | (9.5) | 1 | (reference) | 1 | (reference) |
|  | Widowed | 8,605 | (88.8) | 288 | (3.0) | 799 | (8.2) | 0.74 | (0.66, 0.84) | 0.72 | (0.62, 0.84) |
|  | Divorce/separated | 2,879 | (86.2) | 123 | (3.7) | 336 | (10.1) | 0.98 | (0.82, 1.17) | 0.89 | (0.72, 1.10) |
|  | Never married | 1,831 | (91.1) | 51 | (2.5) | 128 | (6.4) | 0.43 | (0.33, 0.55) | 0.55 | (0.41, 0.73) |
|  | Other | 328 | (91.6) | 6 | (1.7) | 24 | (6.7) | 0.31 | (0.17, 0.57) | 0.32 | (0.16, 0.62) |
| *Cognitive impairment* | |  |  |  |  |  |  |  |  |  |  |
|  | Intact | 9,544 | (86.1) | 457 | (4.1) | 1,086 | (9.8) | 1 | (reference) | 1 | (reference) |
|  | Borderline | 13,251 | (87.1) | 571 | (3.8) | 1,394 | (9.2) | 1.03 | (0.91, 1.15) | 0.86 | (0.76, 0.98) |
|  | Moderately impaired | 7,854 | (88.7) | 298 | (3.4) | 699 | (7.9) | 1.13 | (0.99, 1.30) | 0.78 | (0.66, 0.91) |
|  | Severe + | 1,709 | (88.7) | 44 | (2.3) | 173 | (9.0) | 1.26 | (1.01, 1.57) | 0.64 | (0.49, 0.83) |
| *Pain* | |  |  |  |  |  |  |  |  |  |  |
|  | No pain | 15,011 | (91.5) | 441 | (2.7) | 947 | (5.8) | 1 | (reference) | 1 | (reference) |
|  | Less than daily pain | 6,849 | (87.7) | 344 | (4.4) | 613 | (7.9) | 2.03 | (1.79, 2.30) | 1.41 | (1.23, 1.61) |
|  | Daily pain, not severe | 7,463 | (84.1) | 366 | (4.1) | 1,040 | (11.7) | 3.25 | (2.88, 3.67) | 2.01 | (1.76, 2.29) |
|  | Daily severe pain | 3,040 | (75.8) | 219 | (5.5) | 753 | (18.8) | 8.35 | (7.13, 9.78) | 3.30 | (2.80, 3.90) |
| *Mood* | |  |  |  |  |  |  |  |  |  |  |
|  | Low risk | 20,575 | (90.5) | 660 | (2.9) | 1,499 | (6.6) | 1 | (reference) | 1 | (reference) |
|  | Medium risk | 7,949 | (84.6) | 444 | (4.7) | 1,003 | (10.7) | 2.62 | (2.35, 2.93) | 1.67 | (1.49, 1.87) |
|  | High risk | 3,837 | (77.5) | 266 | (5.4) | 851 | (17.2) | 6.52 | (5.64, 7.54) | 2.49 | (2.15, 2.88) |
| *Smoking status* | |  |  |  |  |  |  |  |  |  |  |
|  | Non-smoker | 30,398 | (87.3) | 1,295 | (3.7) | 3,114 | (8.9) | 1 | (reference) | 1 | (reference) |
|  | Smoker | 1,965 | (86.2) | 75 | (3.3) | 239 | (10.5) | 0.93 | (0.75, 1.15) | 0.82 | (0.66, 1.04) |
| *Alcohol consumption* | |  |  |  |  |  |  |  |  |  |  |
|  | None | 21,881 | (86.6) | 946 | (3.7) | 2,440 | (9.7) | 1 | (reference) | 1 | (reference) |
|  | 1 | 6,027 | (88.8) | 252 | (3.7) | 511 | (7.5) | 0.67 | (0.59, 0.76) | 0.80 | (0.70, 0.92) |
|  | ≥2 | 4,455 | (88.6) | 172 | (3.4) | 402 | (8.0) | 0.59 | (0.51, 0.69) | 0.86 | (0.73, 1.01) |
| *ADL* | |  |  |  |  |  |  |  |  |  |  |
|  | Independent | 20,716 | (88.7) | 772 | (3.3) | 1,861 | (8.0) | 1 | (reference) | 1 | (reference) |
|  | Supervision | 4,462 | (87.6) | 190 | (3.7) | 440 | (8.6) | 1.42 | (1.24, 1.64) | 1.04 | (0.90, 1.21) |
|  | Limited | 3,207 | (83.4) | 181 | (4.7) | 457 | (11.9) | 2.93 | (2.52, 3.41) | 1.34 | (1.14, 1.57) |
|  | Extensive | 2,227 | (83.5) | 126 | (4.7) | 315 | (11.8) | 3.06 | (2.56, 3.66) | 1.35 | (1.11, 1.63) |
|  | Maximal | 791 | (81.5) | 43 | (4.4) | 137 | (14.1) | 4.38 | (3.38, 5.69) | 1.22 | (0.93, 1.60) |
|  | Dependent + | 958 | (82.7) | 58 | (5.0) | 143 | (12.3) | 3.84 | (2.97, 4.97) | 1.07 | (0.81, 1.41) |
| *IADL capacity* | |  |  |  |  |  |  |  |  |  |  |
|  | 0-14 | 8,134 | (91.4) | 227 | (2.6) | 538 | (6.0) | 1 | (reference) | 1 | (reference) |
|  | 15-24 | 7,043 | (87.4) | 301 | (3.7) | 715 | (8.9) | 2.44 | (2.06, 2.89) | 1.50 | (1.28, 1.77) |
|  | 25-34 | 7,980 | (86.1) | 387 | (4.2) | 906 | (9.8) | 3.50 | (2.94, 4.16) | 1.74 | (1.47, 2.07) |
|  | 35-48 | 9,205 | (84.8) | 455 | (4.2) | 1,194 | (11.0) | 5.43 | (4.51, 6.55) | 1.89 | (1.55, 2.31) |
| *Urinary incontinence* | |  |  |  |  |  |  |  |  |  |  |
|  | Continent | 21,637 | (88.7) | 836 | (3.4) | 1,930 | (7.9) | 1 | (reference) | 1 | (reference) |
|  | Occasionally incontinent | 4,729 | (84.9) | 232 | (4.2) | 610 | (10.9) | 2.10 | (1.84, 2.39) | 1.34 | (1.17, 1.53) |
|  | Frequently incontinent | 4,141 | (84.0) | 201 | (4.1) | 587 | (11.9) | 2.94 | (2.55, 3.40) | 1.66 | (1.43, 1.93) |
|  | Otherwise | 1,856 | (85.0) | 101 | (4.6) | 226 | (10.4) | 2.27 | (1.87, 2.76) | 1.12 | (0.92, 1.37) |
| *Medication CAP* | |  |  |  |  |  |  |  |  |  |  |
|  | No | 28,076 | (89.8) | 937 | (3.0) | 2,262 | (7.2) | 1 | (reference) | 1 | (reference) |
|  | Yes | 4,285 | (73.8) | 433 | (7.5) | 1,091 | (18.8) | 5.91 | (5.24, 6.66) | 1.44 | (1.27, 1.64) |
| *Number of disease diagnoses* | |  |  |  |  |  |  |  |  |  |  |
|  | 0 | 4,107 | (91.2) | 110 | (2.4) | 284 | (6.3) | 1 | (reference) | 1 | (reference) |
|  | 1 | 9,811 | (89.7) | 317 | (2.9) | 805 | (7.4) | 1.39 | (1.15, 1.68) | 1.03 | (0.85, 1.25) |
|  | 2 | 9,329 | (87.2) | 429 | (4.0) | 946 | (8.8) | 2.36 | (1.94, 2.87) | 1.23 | (1.01, 1.49) |
|  | 3 | 5,630 | (84.8) | 279 | (4.2) | 731 | (11.0) | 3.54 | (2.88, 4.35) | 1.34 | (1.09, 1.64) |
|  | 4 | 2,381 | (81.5) | 140 | (4.8) | 399 | (13.7) | 6.02 | (4.75, 7.64) | 1.59 | (1.25, 2.01) |
|  | ≥5 | 1,105 | (79.6) | 95 | (6.8) | 188 | (13.5) | 7.00 | (5.27, 9.30) | 1.40 | (1.06, 1.87) |
| *Chest pain* | |  |  |  |  |  |  |  |  |  |  |
|  | Not present | 28,721 | (88.7) | 1,070 | (3.3) | 2,595 | (8.0) | 1 | (reference) | 1 | (reference) |
|  | Present | 3,642 | (77.5) | 300 | (6.4) | 758 | (16.1) | 4.21 | (3.68, 4.83) | 1.37 | (1.20, 1.58) |
| *Difficulty clearing airway secretions* | | |  |  |  |  |  |  |  |  |  |
|  | Not present | 29,381 | (88.9) | 1,085 | (3.3) | 2,589 | (7.8) | 1 | (reference) | 1 | (reference) |
|  | Present | 2,982 | (74.0) | 285 | (7.1) | 764 | (19.0) | 5.95 | (5.11, 6.93) | 1.79 | (1.54, 2.07) |
| *Abnormal thought process* | |  |  |  |  |  |  |  |  |  |  |
|  | Not present | 31,338 | (87.4) | 1,311 | (3.7) | 3,218 | (9.0) | 1 | (reference) | 1 | (reference) |
|  | Present | 1,025 | (84.1) | 59 | (4.8) | 135 | (11.1) | 1.90 | (1.50, 2.40) | 1.31 | (1.00, 1.72) |
| *Delusions* | |  |  |  |  |  |  |  |  |  |  |
|  | Not present | 31,235 | (87.3) | 1,310 | (3.7) | 3,224 | (9.0) | 1 | (reference) | 1 | (reference) |
|  | Present | 1,128 | (85.6) | 60 | (4.6) | 129 | (9.8) | 1.64 | (1.30, 2.06) | 0.97 | (0.74, 1.28) |
| *Hallucinations* | |  |  |  |  |  |  |  |  |  |  |
|  | Not present | 30,819 | (87.7) | 1,256 | (3.6) | 3,049 | (8.7) | 1 | (reference) | 1 | (reference) |
|  | Present | 1,544 | (78.7) | 114 | (5.8) | 304 | (15.5) | 3.48 | (2.88, 4.21) | 2.15 | (1.75, 2.63) |
| *Acid reflux* | |  |  |  |  |  |  |  |  |  |  |
|  | Not present | 27,521 | (88.6) | 1,020 | (3.3) | 2,504 | (8.1) | 1 | (reference) | 1 | (reference) |
|  | Present | 4,842 | (80.2) | 350 | (5.8) | 849 | (14.1) | 3.26 | (2.87, 3.70) | 1.57 | (1.38, 1.79) |
| *Constipation* | |  |  |  |  |  |  |  |  |  |  |
|  | Not present | 25,579 | (89.4) | 861 | (3.0) | 2,158 | (7.5) | 1 | (reference) | 1 | (reference) |
|  | Present | 6,784 | (79.9) | 509 | (6.0) | 1,195 | (14.1) | 4.11 | (3.66, 4.61) | 1.94 | (1.73, 2.18) |
| *Diarrhoea* | |  |  |  |  |  |  |  |  |  |  |
|  | Not present | 30,132 | (88.0) | 1,204 | (3.5) | 2,908 | (8.5) | 1 | (reference) | 1 | (reference) |
|  | Present | 2,231 | (78.5) | 166 | (5.8) | 445 | (15.7) | 3.45 | (2.93, 4.06) | 1.64 | (1.39, 1.93) |
| *Vomiting* | |  |  |  |  |  |  |  |  |  |  |
|  | Not present | 31,833 | (87.5) | 1,315 | (3.6) | 3,212 | (8.8) | 1 | (reference) | 1 | (reference) |
|  | Present | 530 | (73.0) | 55 | (7.6) | 141 | (19.4) | 4.72 | (3.50, 6.35) | 1.50 | (1.09, 2.05) |
| *Aspiration problems* | |  |  |  |  |  |  |  |  |  |  |
|  | Not present | 32,028 | (87.4) | 1,336 | (3.6) | 3,283 | (9.0) | 1 | (reference) | 1 | (reference) |
|  | Present | 335 | (76.3) | 34 | (7.7) | 70 | (15.9) | 4.41 | (3.01, 6.47) | 1.60 | (1.10, 2.35) |
| *Dyspnoea (shortness of breath)* | |  |  |  |  |  |  |  |  |  |  |
|  | Absent | 17,476 | (92.8) | 414 | (2.2) | 946 | (5.0) | 1 | (reference) | 1 | (reference) |
|  | With moderate activities | 7,698 | (88.4) | 324 | (3.7) | 690 | (7.9) | 2.49 | (2.19, 2.83) | 1.73 | (1.51, 1.99) |
|  | With day-to-day activities | 5,093 | (78.6) | 396 | (6.1) | 993 | (15.3) | 9.04 | (7.82, 10.5) | 4.23 | (3.63, 4.92) |
|  | Present at rest | 2,096 | (68.6) | 236 | (7.7) | 724 | (23.7) | 23.1 | (19.1, 28.1) | 7.00 | (5.76, 8.51) |
| *Self-rated health* | |  |  |  |  |  |  |  |  |  |  |
|  | Excellent | 1,193 | (96.4) | 13 | (1.1) | 32 | (2.6) | 1 | (reference) | 1 | (reference) |
|  | Good | 14,295 | (94.1) | 285 | (1.9) | 609 | (4.0) | 1.70 | (1.15, 2.51) | 1.33 | (0.89, 1.98) |
|  | Fair | 10,918 | (85.0) | 598 | (4.7) | 1,327 | (10.3) | 8.60 | (5.82, 12.7) | 3.78 | (2.53, 5.66) |
|  | Poor | 3,162 | (70.5) | 331 | (7.4) | 991 | (22.1) | 36.0 | (23.9, 54.2) | 6.88 | (4.53, 10.5) |
|  | No response | 2,795 | (83.9) | 143 | (4.3) | 394 | (11.8) | 10.6 | (7.08, 15.9) | 4.33 | (2.84, 6.61) |

Note: *Adjusted for age and age^2^; †Adjusted for age, age^2^, loneliness/social isolation, ethnic identification, marital status, cognitive impairment, pain, mood CAP, smoking status, alcohol consumption, ADL, IADL capacity, urinary incontinence, medication CAP, number of disease diagnoses, chest pain, difficulty clearing airway secretions, abnormal thought process, delusions, hallucinations, acid reflux, constipation, diarrhoea, vomiting, aspiration problems, dyspnoea, and self-rated health. In the adjusted analyses, 56 (0.1%) interRAI-HC assessments were omitted due to missing values.

**Table S4**. Distribution of factors by the too much sleep variable for women at first assessment, together with their estimated crude and adjusted odds ratios (ORs) and associated 95% confidence intervals (CIs) in the complete case random effects ordinal logistic regression over all assessments.

|  |  | Too much sleep | | | | | |  | |  | |
| --- | --- | --- | --- | --- | --- | --- | --- | --- | --- | --- | --- |
|  |  | None/minimal | | Moderate | | Severe | | Crude* | | Adjusted† | |
| **Women** | | n | (%) | n | (%) | n | (%) | OR | (95% CI) | OR | (95% CI) |
| *Loneliness/social isolation* | |  |  |  |  |  |  |  |  |  |  |
|  | Not lonely/lives with others | 21,044 | (93.9) | 279 | (1.2) | 1,081 | (4.8) | 1 | (reference) | 1 | (reference) |
|  | Not lonely/lives alone | 21,190 | (96.8) | 183 | (0.8) | 513 | (2.3) | 0.29 | (0.25, 0.33) | 0.72 | (0.60, 0.86) |
|  | Lonely/lives with others | 4,069 | (93.7) | 70 | (1.6) | 202 | (4.7) | 1.04 | (0.86, 1.26) | 0.81 | (0.65, 1.00) |
|  | Lonely/lives alone | 8,950 | (96.1) | 121 | (1.3) | 246 | (2.6) | 0.48 | (0.41, 0.57) | 0.76 | (0.62, 0.94) |
| *Ethnicity* | |  |  |  |  |  |  |  |  |  |  |
|  | European | 48,192 | (95.3) | 585 | (1.2) | 1,794 | (3.5) | 1 | (reference) | 1 | (reference) |
|  | Māori | 3,470 | (95.7) | 37 | (1.0) | 118 | (3.3) | 0.79 | (0.60, 1.02) | 0.45 | (0.34, 0.61) |
|  | Pacific | 1,707 | (95.6) | 16 | (0.9) | 62 | (3.5) | 0.81 | (0.56, 1.17) | 0.35 | (0.23, 0.51) |
|  | Other | 1,889 | (95.7) | 15 | (0.8) | 70 | (3.5) | 0.77 | (0.54, 1.10) | 0.37 | (0.25, 0.54) |
| *Marital status* | |  |  |  |  |  |  |  |  |  |  |
|  | Married/de facto | 15,914 | (94.4) | 223 | (1.3) | 716 | (4.2) | 1 | (reference) | 1 | (reference) |
|  | Widowed | 33,191 | (95.7) | 353 | (1.0) | 1,121 | (3.2) | 0.55 | (0.47, 0.63) | 0.79 | (0.66, 0.93) |
|  | Divorce/separated | 3,726 | (95.3) | 50 | (1.3) | 135 | (3.5) | 0.64 | (0.50, 0.83) | 0.86 | (0.64, 1.16) |
|  | Never married | 1,962 | (96.0) | 23 | (1.1) | 58 | (2.8) | 0.38 | (0.26, 0.56) | 0.76 | (0.50, 1.15) |
|  | Other | 430 | (96.0) | 4 | (0.9) | 14 | (3.1) | 0.40 | (0.17, 0.93) | 0.71 | (0.29, 1.76) |
| *Cognitive impairment* | |  |  |  |  |  |  |  |  |  |  |
|  | Intact | 21,017 | (97.2) | 194 | (0.9) | 420 | (1.9) | 1 | (reference) | 1 | (reference) |
|  | Borderline | 21,521 | (95.3) | 268 | (1.2) | 802 | (3.6) | 2.60 | (2.20, 3.08) | 1.92 | (1.62, 2.27) |
|  | Moderately impaired | 10,448 | (93.6) | 157 | (1.4) | 552 | (4.9) | 5.59 | (4.58, 6.84) | 2.32 | (1.89, 2.85) |
|  | Severe + | 2,266 | (88.2) | 34 | (1.3) | 268 | (10.4) | 17.5 | (13.7, 22.5) | 2.86 | (2.13, 3.83) |
| *Pain* | |  |  |  |  |  |  |  |  |  |  |
|  | No pain | 20,079 | (96.1) | 182 | (0.9) | 635 | (3.0) | 1 | (reference) | 1 | (reference) |
|  | Less than daily pain | 12,062 | (95.6) | 143 | (1.1) | 406 | (3.2) | 1.30 | (1.11, 1.52) | 1.16 | (0.97, 1.38) |
|  | Daily pain, not severe | 15,212 | (95.1) | 190 | (1.2) | 597 | (3.7) | 1.59 | (1.38, 1.84) | 1.39 | (1.18, 1.64) |
|  | Daily severe pain | 7,905 | (93.6) | 138 | (1.6) | 406 | (4.8) | 2.66 | (2.24, 3.15) | 1.75 | (1.43, 2.14) |
| *Mood* | |  |  |  |  |  |  |  |  |  |  |
|  | Low risk | 33,840 | (96.8) | 244 | (0.7) | 891 | (2.5) | 1 | (reference) | 1 | (reference) |
|  | Medium risk | 13,261 | (94.4) | 203 | (1.4) | 585 | (4.2) | 2.66 | (2.31, 3.07) | 1.59 | (1.37, 1.84) |
|  | High risk | 8,155 | (91.3) | 206 | (2.3) | 568 | (6.4) | 6.10 | (5.21, 7.15) | 2.30 | (1.94, 2.73) |
| *Smoking status* | |  |  |  |  |  |  |  |  |  |  |
|  | Non-smoker | 52,498 | (95.4) | 607 | (1.1) | 1,912 | (3.5) | 1 | (reference) | 1 | (reference) |
|  | Smoker | 2,760 | (93.9) | 46 | (1.6) | 132 | (4.5) | 1.31 | (1.01, 1.70) | 1.42 | (1.06, 1.90) |
| *Alcohol consumption* | |  |  |  |  |  |  |  |  |  |  |
|  | None | 45,316 | (95.1) | 550 | (1.2) | 1,761 | (3.7) | 1 | (reference) | 1 | (reference) |
|  | 1 | 7,259 | (96.1) | 77 | (1.0) | 218 | (2.9) | 0.64 | (0.53, 0.76) | 0.97 | (0.79, 1.19) |
|  | ≥2 | 2,683 | (96.7) | 26 | (0.9) | 65 | (2.3) | 0.51 | (0.37, 0.70) | 0.80 | (0.57, 1.12) |
| *ADL* | |  |  |  |  |  |  |  |  |  |  |
|  | Independent | 38,399 | (97.0) | 333 | (0.8) | 856 | (2.2) | 1 | (reference) | 1 | (reference) |
|  | Supervision | 6,200 | (94.4) | 99 | (1.5) | 268 | (4.1) | 2.91 | (2.44, 3.47) | 1.12 | (0.93, 1.36) |
|  | Limited | 5,013 | (92.0) | 113 | (2.1) | 323 | (5.9) | 5.70 | (4.76, 6.81) | 1.62 | (1.35, 1.96) |
|  | Extensive | 2,979 | (92.0) | 61 | (1.9) | 199 | (6.1) | 7.30 | (5.94, 8.98) | 1.50 | (1.20, 1.89) |
|  | Maximal | 1,092 | (86.4) | 24 | (1.9) | 148 | (11.7) | 19.7 | (15.1, 25.7) | 2.67 | (1.99, 3.57) |
|  | Dependent + | 1,572 | (85.2) | 23 | (1.2) | 250 | (13.6) | 21.9 | (17.2, 27.9) | 3.01 | (2.30, 3.95) |
| *IADL capacity* | |  |  |  |  |  |  |  |  |  |  |
|  | 0-14 | 15,132 | (98.3) | 99 | (0.6) | 159 | (1.0) | 1 | (reference) | 1 | (reference) |
|  | 15-24 | 14,320 | (96.9) | 146 | (1.0) | 311 | (2.1) | 3.01 | (2.38, 3.81) | 1.85 | (1.47, 2.34) |
|  | 25-34 | 13,626 | (95.3) | 195 | (1.4) | 478 | (3.3) | 7.64 | (5.81, 10.0) | 2.94 | (2.31, 3.75) |
|  | 35-48 | 12,177 | (90.3) | 213 | (1.6) | 1,096 | (8.1) | 34.9 | (25.7, 47.5) | 6.36 | (4.81, 8.41) |
| *Urinary incontinence* | |  |  |  |  |  |  |  |  |  |  |
|  | Continent | 32,750 | (96.5) | 307 | (0.9) | 864 | (2.5) | 1 | (reference) | 1 | (reference) |
|  | Occasionally incontinent | 11,173 | (95.0) | 167 | (1.4) | 427 | (3.6) | 2.01 | (1.72, 2.36) | 1.07 | (0.91, 1.26) |
|  | Frequently incontinent | 10,704 | (92.5) | 165 | (1.4) | 708 | (6.1) | 5.13 | (4.39, 5.99) | 1.54 | (1.32, 1.80) |
|  | Otherwise | 631 | (91.4) | 14 | (2.0) | 45 | (6.5) | 6.07 | (4.03, 9.14) | 1.21 | (0.78, 1.89) |
| *Medication CAP* | |  |  |  |  |  |  |  |  |  |  |
|  | No | 47,008 | (96.0) | 463 | (0.9) | 1,500 | (3.1) | 1 | (reference) | 1 | (reference) |
|  | Yes | 8,247 | (91.8) | 190 | (2.1) | 544 | (6.1) | 3.38 | (2.97, 3.84) | 1.32 | (1.13, 1.55) |
| *Number of disease diagnoses* | |  |  |  |  |  |  |  |  |  |  |
|  | 0 | 10,802 | (97.5) | 69 | (0.6) | 206 | (1.9) | 1 | (reference) | 1 | (reference) |
|  | 1 | 17,983 | (96.2) | 173 | (0.9) | 542 | (2.9) | 2.26 | (1.82, 2.79) | 1.32 | (1.06, 1.65) |
|  | 2 | 14,363 | (95.0) | 158 | (1.0) | 603 | (4.0) | 3.62 | (2.89, 4.53) | 1.47 | (1.17, 1.84) |
|  | 3 | 7,689 | (93.8) | 132 | (1.6) | 374 | (4.6) | 5.97 | (4.66, 7.63) | 1.68 | (1.31, 2.15) |
|  | 4 | 3,108 | (91.7) | 73 | (2.2) | 208 | (6.1) | 10.0 | (7.59, 13.2) | 2.09 | (1.58, 2.78) |
|  | ≥5 | 1,313 | (89.2) | 48 | (3.3) | 111 | (7.5) | 17.0 | (12.3, 23.6) | 2.37 | (1.68, 3.33) |
| *Chest pain* | |  |  |  |  |  |  |  |  |  |  |
|  | Not present | 49,175 | (95.7) | 538 | (1.0) | 1,691 | (3.3) | 1 | (reference) | 1 | (reference) |
|  | Present | 6,083 | (92.9) | 115 | (1.8) | 353 | (5.4) | 2.27 | (1.94, 2.65) | 1.34 | (1.12, 1.62) |
| *Difficulty clearing airway secretions* | | |  |  |  |  |  |  |  |  |  |
|  | Not present | 51,373 | (95.7) | 562 | (1.0) | 1,735 | (3.2) | 1 | (reference) | 1 | (reference) |
|  | Present | 3,885 | (90.7) | 91 | (2.1) | 309 | (7.2) | 3.66 | (3.08, 4.35) | 1.55 | (1.26, 1.90) |
| *Abnormal thought process* | |  |  |  |  |  |  |  |  |  |  |
|  | Not present | 53,705 | (95.5) | 620 | (1.1) | 1,927 | (3.4) | 1 | (reference) | 1 | (reference) |
|  | Present | 1,553 | (91.2) | 33 | (1.9) | 117 | (6.9) | 3.54 | (2.78, 4.51) | 1.58 | (1.17, 2.14) |
| *Delusions* | |  |  |  |  |  |  |  |  |  |  |
|  | Not present | 53,376 | (95.4) | 624 | (1.1) | 1,950 | (3.5) | 1 | (reference) | 1 | (reference) |
|  | Present | 1,882 | (93.9) | 29 | (1.4) | 94 | (4.7) | 1.98 | (1.54, 2.55) | 0.70 | (0.51, 0.96) |
| *Hallucinations* | |  |  |  |  |  |  |  |  |  |  |
|  | Not present | 53,069 | (95.5) | 613 | (1.1) | 1,867 | (3.4) | 1 | (reference) | 1 | (reference) |
|  | Present | 2,189 | (91.0) | 40 | (1.7) | 177 | (7.4) | 3.59 | (2.93, 4.40) | 1.41 | (1.10, 1.81) |
| *Acid reflux* | |  |  |  |  |  |  |  |  |  |  |
|  | Not present | 45,475 | (95.7) | 491 | (1.0) | 1,577 | (3.3) | 1 | (reference) | 1 | (reference) |
|  | Present | 9,783 | (94.0) | 162 | (1.6) | 467 | (4.5) | 1.91 | (1.67, 2.20) | 1.30 | (1.11, 1.52) |
| *Constipation* | |  |  |  |  |  |  |  |  |  |  |
|  | Not present | 43,581 | (96.0) | 449 | (1.0) | 1,351 | (3.0) | 1 | (reference) | 1 | (reference) |
|  | Present | 11,677 | (92.9) | 204 | (1.6) | 693 | (5.5) | 3.09 | (2.72, 3.52) | 1.73 | (1.50, 1.98) |
| *Diarrhoea* | |  |  |  |  |  |  |  |  |  |  |
|  | Not present | 50,902 | (95.7) | 545 | (1.0) | 1,767 | (3.3) | 1 | (reference) | 1 | (reference) |
|  | Present | 4,356 | (91.9) | 108 | (2.3) | 277 | (5.8) | 3.21 | (2.72, 3.77) | 1.78 | (1.47, 2.14) |
| *Vomiting* | |  |  |  |  |  |  |  |  |  |  |
|  | Not present | 54,045 | (95.5) | 624 | (1.1) | 1,905 | (3.4) | 1 | (reference) | 1 | (reference) |
|  | Present | 1,213 | (87.8) | 29 | (2.1) | 139 | (10.1) | 5.77 | (4.44, 7.50) | 2.21 | (1.63, 3.00) |
| *Aspiration problems* | |  |  |  |  |  |  |  |  |  |  |
|  | Not present | 54,869 | (95.4) | 641 | (1.1) | 2,002 | (3.5) | 1 | (reference) | 1 | (reference) |
|  | Present | 389 | (87.8) | 12 | (2.7) | 42 | (9.5) | 4.88 | (3.09, 7.72) | 1.36 | (0.80, 2.30) |
| *Dyspnoea (shortness of breath)* | |  |  |  |  |  |  |  |  |  |  |
|  | Absent | 29,960 | (96.7) | 220 | (0.7) | 798 | (2.6) | 1 | (reference) | 1 | (reference) |
|  | With moderate activities | 13,048 | (95.4) | 176 | (1.3) | 447 | (3.3) | 1.67 | (1.44, 1.95) | 1.70 | (1.44, 2.02) |
|  | With day-to-day activities | 9,052 | (93.1) | 172 | (1.8) | 494 | (5.1) | 3.68 | (3.13, 4.33) | 2.90 | (2.40, 3.49) |
|  | Present at rest | 3,198 | (89.1) | 85 | (2.4) | 305 | (8.5) | 8.51 | (6.93, 10.4) | 4.24 | (3.31, 5.43) |
| *Self-rated health* | |  |  |  |  |  |  |  |  |  |  |
|  | Excellent | 1,946 | (98.1) | 11 | (0.6) | 26 | (1.3) | 1 | (reference) | 1 | (reference) |
|  | Good | 25,309 | (97.7) | 149 | (0.6) | 435 | (1.7) | 1.35 | (0.89, 2.07) | 1.21 | (0.77, 1.91) |
|  | Fair | 19,032 | (95.2) | 294 | (1.5) | 665 | (3.3) | 4.99 | (3.23, 7.71) | 2.83 | (1.78, 4.49) |
|  | Poor | 5,306 | (90.1) | 127 | (2.2) | 457 | (7.8) | 18.9 | (12.0, 29.8) | 5.30 | (3.26, 8.62) |
|  | No response | 3,665 | (87.3) | 72 | (1.7) | 461 | (11.0) | 27.7 | (17.5, 43.9) | 5.77 | (3.54, 9.40) |

Note: *Adjusted for age and age^2^; †Adjusted for age, age^2^, loneliness/social isolation, ethnic identification, marital status, cognitive impairment, pain, mood CAP, smoking status, alcohol consumption, ADL, IADL capacity, urinary incontinence, medication CAP, number of disease diagnoses, chest pain, difficulty clearing airway secretions, abnormal thought process, delusions, hallucinations, acid reflux, constipation, diarrhoea, vomiting, aspiration problems, dyspnoea, and self-rated health. In the adjusted analyses, 82 (0.09%) interRAI-HC assessments were omitted due to missing values.

**Table S5**. Distribution of factors by the too much sleep variable for men at first assessment, together with their estimated crude and adjusted odds ratios (ORs) and associated 95% confidence intervals (CIs) in the complete case random effects ordinal logistic regression over all assessments.

|  |  | Too much sleep | | | | | |  | |  | |
| --- | --- | --- | --- | --- | --- | --- | --- | --- | --- | --- | --- |
|  |  | None/minimal | | Moderate | | Severe | | Crude* | | Adjusted† | |
| **Men** | | n | (%) | n | (%) | n | (%) | OR | (95% CI) | OR | (95% CI) |
| *Loneliness/social isolation* | |  |  |  |  |  |  |  |  |  |  |
|  | Not lonely/lives with others | 19,625 | (90.9) | 392 | (1.8) | 1,578 | (7.3) | 1 | (reference) | 1 | (reference) |
|  | Not lonely/lives alone | 8,107 | (95.8) | 101 | (1.2) | 255 | (3.0) | 0.23 | (0.19, 0.29) | 0.63 | (0.50, 0.80) |
|  | Lonely/lives with others | 2,659 | (90.1) | 62 | (2.1) | 231 | (7.8) | 1.19 | (0.98, 1.44) | 0.87 | (0.69, 1.10) |
|  | Lonely/lives alone | 3,873 | (95.2) | 59 | (1.4) | 138 | (3.4) | 0.30 | (0.24, 0.38) | 0.59 | (0.45, 0.79) |
| *Ethnicity* | |  |  |  |  |  |  |  |  |  |  |
|  | European | 30,026 | (92.2) | 530 | (1.6) | 2,009 | (6.2) | 1 | (reference) | 1 | (reference) |
|  | Māori | 1,972 | (94.2) | 43 | (2.1) | 79 | (3.8) | 0.52 | (0.37, 0.74) | 0.35 | (0.24, 0.50) |
|  | Pacific | 1,085 | (94.3) | 17 | (1.5) | 48 | (4.2) | 0.46 | (0.29, 0.73) | 0.20 | (0.12, 0.32) |
|  | Other | 1,186 | (92.9) | 24 | (1.9) | 67 | (5.2) | 0.89 | (0.62, 1.26) | 0.47 | (0.32, 0.70) |
| *Marital status* | |  |  |  |  |  |  |  |  |  |  |
|  | Married/de facto | 19,695 | (90.9) | 398 | (1.8) | 1,567 | (7.2) | 1 | (reference) | 1 | (reference) |
|  | Widowed | 9,151 | (94.4) | 131 | (1.4) | 410 | (4.2) | 0.41 | (0.35, 0.48) | 0.76 | (0.62, 0.93) |
|  | Divorce/separated | 3,136 | (93.9) | 65 | (1.9) | 137 | (4.1) | 0.43 | (0.33, 0.55) | 0.89 | (0.66, 1.20) |
|  | Never married | 1,920 | (95.5) | 16 | (0.8) | 74 | (3.7) | 0.25 | (0.17, 0.38) | 0.55 | (0.36, 0.83) |
|  | Other | 341 | (95.3) | 4 | (1.1) | 13 | (3.6) | 0.27 | (0.11, 0.64) | 0.40 | (0.16, 0.98) |
| *Cognitive impairment* | |  |  |  |  |  |  |  |  |  |  |
|  | Intact | 10,544 | (95.1) | 146 | (1.3) | 397 | (3.6) | 1 | (reference) | 1 | (reference) |
|  | Borderline | 14,190 | (93.3) | 232 | (1.5) | 794 | (5.2) | 3.10 | (2.37, 4.06) | 1.66 | (1.37, 2.02) |
|  | Moderately impaired | 7,932 | (89.6) | 192 | (2.2) | 727 | (8.2) | 12.1 | (9.27, 15.7) | 2.70 | (2.14, 3.40) |
|  | Severe + | 1,598 | (83.0) | 44 | (2.3) | 284 | (14.7) | 39.3 | (28.0, 55.2) | 2.82 | (2.05, 3.88) |
| *Pain* | |  |  |  |  |  |  |  |  |  |  |
|  | No pain | 15,340 | (93.5) | 223 | (1.4) | 836 | (5.1) | 1 | (reference) | 1 | (reference) |
|  | Less than daily pain | 7,222 | (92.5) | 152 | (1.9) | 432 | (5.5) | 1.35 | (1.14, 1.59) | 1.14 | (0.96, 1.36) |
|  | Daily pain, not severe | 8,172 | (92.1) | 153 | (1.7) | 544 | (6.1) | 1.49 | (1.27, 1.74) | 1.25 | (1.04, 1.48) |
|  | Daily severe pain | 3,535 | (88.1) | 86 | (2.1) | 391 | (9.7) | 3.53 | (2.88, 4.33) | 2.16 | (1.71, 2.71) |
| *Mood* | |  |  |  |  |  |  |  |  |  |  |
|  | Low risk | 21,508 | (94.6) | 262 | (1.2) | 964 | (4.2) | 1 | (reference) | 1 | (reference) |
|  | Medium risk | 8,521 | (90.7) | 197 | (2.1) | 678 | (7.2) | 2.90 | (2.49, 3.37) | 1.69 | (1.45, 1.97) |
|  | High risk | 4,238 | (85.5) | 155 | (3.1) | 561 | (11.3) | 6.66 | (5.52, 8.05) | 2.39 | (1.97, 2.90) |
| *Smoking status* | |  |  |  |  |  |  |  |  |  |  |
|  | Non-smoker | 32,147 | (92.4) | 580 | (1.7) | 2,080 | (6.0) | 1 | (reference) | 1 | (reference) |
|  | Smoker | 2,122 | (93.1) | 34 | (1.5) | 123 | (5.4) | 0.74 | (0.55, 0.99) | 0.93 | (0.68, 1.28) |
| *Alcohol consumption* | |  |  |  |  |  |  |  |  |  |  |
|  | None | 23,148 | (91.6) | 453 | (1.8) | 1,666 | (6.6) | 1 | (reference) | 1 | (reference) |
|  | 1 | 6,360 | (93.7) | 100 | (1.5) | 330 | (4.9) | 0.51 | (0.43, 0.62) | 0.79 | (0.65, 0.95) |
|  | ≥2 | 4,761 | (94.7) | 61 | (1.2) | 207 | (4.1) | 0.36 | (0.28, 0.46) | 0.77 | (0.61, 0.97) |
| *ADL* | |  |  |  |  |  |  |  |  |  |  |
|  | Independent | 22,271 | (95.4) | 274 | (1.2) | 804 | (3.4) | 1 | (reference) | 1 | (reference) |
|  | Supervision | 4,604 | (90.4) | 115 | (2.3) | 373 | (7.3) | 3.64 | (3.03, 4.37) | 1.40 | (1.15, 1.71) |
|  | Limited | 3,411 | (88.7) | 89 | (2.3) | 345 | (9.0) | 6.18 | (5.08, 7.53) | 1.56 | (1.26, 1.93) |
|  | Extensive | 2,288 | (85.8) | 68 | (2.5) | 312 | (11.7) | 9.81 | (7.86, 12.3) | 2.05 | (1.62, 2.59) |
|  | Maximal | 789 | (81.3) | 32 | (3.3) | 150 | (15.4) | 19.0 | (14.0, 25.8) | 2.31 | (1.69, 3.18) |
|  | Dependent + | 904 | (78.0) | 36 | (3.1) | 219 | (18.9) | 30.7 | (22.6, 41.8) | 3.93 | (2.85, 5.43) |
| *IADL capacity* | |  |  |  |  |  |  |  |  |  |  |
|  | 0-14 | 8,719 | (98.0) | 57 | (0.6) | 123 | (1.4) | 1 | (reference) | 1 | (reference) |
|  | 15-24 | 7,669 | (95.2) | 114 | (1.4) | 276 | (3.4) | 4.82 | (3.34, 6.96) | 2.41 | (1.80, 3.23) |
|  | 25-34 | 8,580 | (92.5) | 176 | (1.9) | 517 | (5.6) | 12.8 | (8.48, 19.3) | 4.03 | (2.99, 5.45) |
|  | 35-48 | 9,300 | (85.7) | 267 | (2.5) | 1,287 | (11.9) | 58.8 | (36.9, 93.6) | 7.51 | (5.37, 10.5) |
| *Urinary incontinence* | |  |  |  |  |  |  |  |  |  |  |
|  | Continent | 22,996 | (94.2) | 329 | (1.3) | 1,078 | (4.4) | 1 | (reference) | 1 | (reference) |
|  | Occasionally incontinent | 5,038 | (90.4) | 129 | (2.3) | 404 | (7.3) | 2.69 | (2.27, 3.19) | 1.23 | (1.03, 1.47) |
|  | Frequently incontinent | 4,283 | (86.9) | 109 | (2.2) | 537 | (10.9) | 6.12 | (5.03, 7.45) | 1.51 | (1.25, 1.82) |
|  | Otherwise | 1,952 | (89.4) | 47 | (2.2) | 184 | (8.4) | 4.23 | (3.29, 5.44) | 1.28 | (0.98, 1.67) |
| *Medication CAP* | |  |  |  |  |  |  |  |  |  |  |
|  | No | 29,195 | (93.3) | 454 | (1.5) | 1,626 | (5.2) | 1 | (reference) | 1 | (reference) |
|  | Yes | 5,072 | (87.3) | 160 | (2.8) | 577 | (9.9) | 3.27 | (2.81, 3.80) | 1.24 | (1.04, 1.49) |
| *Number of disease diagnoses* | |  |  |  |  |  |  |  |  |  |  |
|  | 0 | 4,311 | (95.8) | 36 | (0.8) | 154 | (3.4) | 1 | (reference) | 1 | (reference) |
|  | 1 | 10,246 | (93.7) | 142 | (1.3) | 545 | (5.0) | 3.23 | (2.01, 5.18) | 1.47 | (1.11, 1.96) |
|  | 2 | 9,860 | (92.1) | 174 | (1.6) | 670 | (6.3) | 5.31 | (3.34, 8.46) | 1.63 | (1.23, 2.17) |
|  | 3 | 6,009 | (90.5) | 143 | (2.2) | 488 | (7.3) | 10.8 | (6.91, 17.0) | 1.95 | (1.45, 2.63) |
|  | 4 | 2,621 | (89.8) | 70 | (2.4) | 229 | (7.8) | 17.1 | (10.8, 27.1) | 1.89 | (1.36, 2.64) |
|  | ≥5 | 1,222 | (88.0) | 49 | (3.5) | 117 | (8.4) | 26.3 | (16.1, 42.8) | 1.84 | (1.25, 2.71) |
| *Chest pain* | |  |  |  |  |  |  |  |  |  |  |
|  | Not present | 30,062 | (92.8) | 489 | (1.5) | 1,835 | (5.7) | 1 | (reference) | 1 | (reference) |
|  | Present | 4,207 | (89.5) | 125 | (2.7) | 368 | (7.8) | 1.94 | (1.64, 2.30) | 0.96 | (0.79, 1.17) |
| *Difficulty clearing airway secretions* | | |  |  |  |  |  |  |  |  |  |
|  | Not present | 30,830 | (93.3) | 475 | (1.4) | 1,750 | (5.3) | 1 | (reference) | 1 | (reference) |
|  | Present | 3,439 | (85.3) | 139 | (3.4) | 453 | (11.2) | 4.36 | (3.65, 5.21) | 1.95 | (1.60, 2.38) |
| *Abnormal thought process* | |  |  |  |  |  |  |  |  |  |  |
|  | Not present | 33,196 | (92.6) | 580 | (1.6) | 2,091 | (5.8) | 1 | (reference) | 1 | (reference) |
|  | Present | 1,073 | (88.0) | 34 | (2.8) | 112 | (9.2) | 3.06 | (2.29, 4.07) | 1.15 | (0.81, 1.62) |
| *Delusions* | |  |  |  |  |  |  |  |  |  |  |
|  | Not present | 33,100 | (92.5) | 580 | (1.6) | 2,089 | (5.8) | 1 | (reference) | 1 | (reference) |
|  | Present | 1,169 | (88.8) | 34 | (2.6) | 114 | (8.7) | 2.54 | (1.92, 3.36) | 0.87 | (0.63, 1.21) |
| *Hallucinations* | |  |  |  |  |  |  |  |  |  |  |
|  | Not present | 32,586 | (92.8) | 552 | (1.6) | 1,986 | (5.7) | 1 | (reference) | 1 | (reference) |
|  | Present | 1,683 | (85.8) | 62 | (3.2) | 217 | (11.1) | 3.75 | (3.00, 4.68) | 1.27 | (0.99, 1.62) |
| *Acid reflux* | |  |  |  |  |  |  |  |  |  |  |
|  | Not present | 28,854 | (92.9) | 484 | (1.6) | 1,707 | (5.5) | 1 | (reference) | 1 | (reference) |
|  | Present | 5,415 | (89.6) | 130 | (2.2) | 496 | (8.2) | 1.98 | (1.69, 2.32) | 1.36 | (1.14, 1.63) |
| *Constipation* | |  |  |  |  |  |  |  |  |  |  |
|  | Not present | 26,734 | (93.5) | 414 | (1.4) | 1,450 | (5.1) | 1 | (reference) | 1 | (reference) |
|  | Present | 7,535 | (88.8) | 200 | (2.4) | 753 | (8.9) | 2.95 | (2.52, 3.44) | 1.50 | (1.28, 1.75) |
| *Diarrhoea* | |  |  |  |  |  |  |  |  |  |  |
|  | Not present | 31,802 | (92.9) | 526 | (1.5) | 1,916 | (5.6) | 1 | (reference) | 1 | (reference) |
|  | Present | 2,467 | (86.8) | 88 | (3.1) | 287 | (10.1) | 3.37 | (2.75, 4.13) | 1.87 | (1.51, 2.32) |
| *Vomiting* | |  |  |  |  |  |  |  |  |  |  |
|  | Not present | 33,684 | (92.6) | 580 | (1.6) | 2,096 | (5.8) | 1 | (reference) | 1 | (reference) |
|  | Present | 585 | (80.6) | 34 | (4.7) | 107 | (14.7) | 6.93 | (4.86, 9.89) | 2.57 | (1.76, 3.75) |
| *Aspiration problems* | |  |  |  |  |  |  |  |  |  |  |
|  | Not present | 33,894 | (92.5) | 596 | (1.6) | 2,157 | (5.9) | 1 | (reference) | 1 | (reference) |
|  | Present | 375 | (85.4) | 18 | (4.1) | 46 | (10.5) | 4.08 | (2.64, 6.31) | 1.04 | (0.64, 1.68) |
| *Dyspnoea (shortness of breath)* | |  |  |  |  |  |  |  |  |  |  |
|  | Absent | 17,783 | (94.4) | 208 | (1.1) | 845 | (4.5) | 1 | (reference) | 1 | (reference) |
|  | With moderate activities | 8,116 | (93.2) | 144 | (1.7) | 452 | (5.2) | 1.45 | (1.23, 1.72) | 1.46 | (1.21, 1.75) |
|  | With day-to-day activities | 5,777 | (89.1) | 172 | (2.7) | 533 | (8.2) | 3.72 | (3.12, 4.44) | 2.96 | (2.41, 3.62) |
|  | Present at rest | 2,593 | (84.8) | 90 | (2.9) | 373 | (12.2) | 7.92 | (6.25, 10.0) | 3.85 | (2.97, 5.00) |
| *Self-rated health* | |  |  |  |  |  |  |  |  |  |  |
|  | Excellent | 1,203 | (97.2) | 7 | (0.6) | 28 | (2.3) | 1 | (reference) | 1 | (reference) |
|  | Good | 14,618 | (96.2) | 118 | (0.8) | 453 | (3.0) | 1.73 | (1.04, 2.89) | 1.53 | (0.90, 2.61) |
|  | Fair | 11,881 | (92.5) | 250 | (1.9) | 712 | (5.5) | 5.86 | (3.50, 9.82) | 3.47 | (2.02, 5.94) |
|  | Poor | 3,784 | (84.4) | 158 | (3.5) | 542 | (12.1) | 25.1 | (14.7, 42.9) | 7.06 | (4.03, 12.4) |
|  | No response | 2,783 | (83.5) | 81 | (2.4) | 468 | (14.0) | 30.9 | (17.9, 53.1) | 6.54 | (3.75, 11.4) |

Note: *Adjusted for age; †Adjusted for age, loneliness/social isolation, ethnic identification, marital status, cognitive impairment, pain, mood CAP, smoking status, alcohol consumption, ADL, IADL capacity, urinary incontinence, medication CAP, number of disease diagnoses, chest pain, difficulty clearing airway secretions, abnormal thought process, delusions, hallucinations, acid reflux, constipation, diarrhoea, vomiting, aspiration problems, dyspnoea, and self-rated health. In the adjusted analyses, 56 (0.1%) interRAI-HC assessments were omitted due to missing values.
